# Seasonal Changes in Socio-Spatial Structure in a Group of Free-Living Spider Monkeys (*Ateles geoffroyi*)

**DOI:** 10.1371/journal.pone.0157228

**Published:** 2016-06-09

**Authors:** Sandra E. Smith-Aguilar, Gabriel Ramos-Fernández, Wayne M. Getz

**Affiliations:** 1 Instituto Politécnico Nacional, Centro Interdisciplinario de Investigación para el Desarrollo Integral Regional Unidad Oaxaca, Santa Cruz Xoxocotlán, Oaxaca, México; 2 Centro de Ciencias de la Complejidad, Universidad Nacional Autónoma de México, Ciudad de México, México; 3 Department of Environmental Science, Policy and Management, University of California, Berkeley, California, United States of America; 4 School of Mathematical Sciences, University of KwaZulu-Natal, Durban, South Africa; Centre for Ecological and Evolutionary Studies, NETHERLANDS

## Abstract

Ecological and social factors influence individual movement and group membership decisions, which ultimately determine how animal groups adjust their behavior in spatially and temporally heterogeneous environments. The mechanisms behind these behavioral adjustments can be better understood by studying the relationship between association and space use patterns of groups and how these change over time. We examined the socio-spatial patterns of adult individuals in a free-ranging group of spider monkeys (*Ateles geoffroyi*), a species with high fission-fusion dynamics. Data comprised 4916 subgroup scans collected during 325 days throughout a 20-month period and was used to evaluate changes from fruit-scarce to fruit-abundant periods in individual core-area size, subgroup size and two types of association measures: spatial (core-area overlap) and spatio-temporal (occurrence in the same subgroup) associations. We developed a 3-level analysis framework to distinguish passive associations, where individuals are mostly brought together by resources of common interest, from active association, where individuals actively seek or avoid certain others. Results indicated a more concentrated use of space, increased individual gregariousness and higher spatio-temporal association rates in the fruit-abundant seasons, as is compatible with an increase in passive associations. Nevertheless, results also suggested active associations in all the periods analyzed, although associations differed across seasons. In particular, females seem to actively avoid males, perhaps prompted by an increased probability of random encounters among individuals, resulting from the contraction of individual core areas. Our framework proved useful in investigating the interplay between ecological and social constraints and how these constraints can influence individual ranging and grouping decisions in spider monkeys, and possibly other species with high fission-fusion dynamics.

## Introduction

Ecological factors, particularly the distribution and abundance of food and the risk of predation, have long been recognized as important drivers of animal space use and social organization [1–6]. More recent evidence also indicates that the spatial distribution of group members may shape competitive, cooperative, and dominance patterns of interaction or relationships, as well as mechanisms for information transfer [7–12]. Modeling studies have shown that individuals interacting in certain spatial configurations can develop emergent social relationships, such as reciprocation [13,14]. Analyses of empirical data have documented direct fitness costs and benefits conferred by social relationships [15–17]. We only have a scant understanding, however, of how ecological and social factors interact to determine the short-term movement and grouping decisions of individuals (though see: [18–22]). Ramos-Fernández and Morales [23] suggested that rules of social engagement can function as mediating mechanisms through which ecologically-dependent processes operate on a short-term basis (see also: [24–26]).

The result of the interdependence between spatial and social influences on social organization is recognized as the socio-spatial structure of groups [14,27–29]. Fission-fusion dynamics are an example of how animals adjust their socio-spatial structure to changing environmental conditions, presumably as a way to balance the costs and benefits of group-living [5,30–33]. Groups that constantly vary in size, composition and cohesion are deemed as having high fission-fusion dynamics and are found precisely in those species that depend on highly unpredictable resources or which show significant periodic changes in abundance and distribution (*e*.*g*. chimpanzees, *Pan troglodytes* and spider monkeys, *Ateles* spp. [34]; bottlenose dolphins, *Tursiops truncatus* [35]; spotted hyenas, *Crocuta crocuta* [36]; African buffalo, *Syncerus caffer* [37] and several bats like *Myotis bechsteinii* [38] and *Nyctalus lasiopterus* [39]). In this plastic social arrangement, grouping and ranging patterns change continuously over time [12,40–42]. This variation has been observed as seasonal changes in average subgroup size [36,43], subgroup cohesion [44,45], subgroup composition [46], intensity and stability of associations [47], movement patterns [48,49] and ranging area [50,51].

Although temporal variation in these features of fission-fusion dynamics has often been found to correlate with resource availability [6,36,52,53], ecological models alone have proven insufficient to explain many of these observations [54–57]. A growing body of evidence suggests that demographic and social factors interact with ecological drivers in determining the spatial arrangement of group members [20,41,50,58–61]. Yet, within this potentially complex synergy of influences [12,13,23,62–65], grouping and ranging patterns in high fission-fusion dynamics species are ultimately the result of individual decisions to join, leave or remain in a certain subgroup [25,66]. Therefore, the co-occurrence of individuals in subgroups (spatio-temporal association) encompasses these individual decisions and their underlying influences [20,67].

Spatio-temporal associations can simply reflect common environmental requirements and preferences, including potential preference for groups themselves or for conspecifics in general (passive association; [22,63,68,69]). These associations may also result from active attraction or repulsion between particular individuals (active associations; [12,70–72]). In the former case, spatio-temporal associations are expected to be similar among all members of the group, varying in the same way and reflecting mostly shifts in resource abundance and distribution. As subgroup sizes increase, every group-member is similarly prone to be a part of larger subgroups (assuming they all use similar areas) and therefore co-occur with more individuals. Increasing the average number of subgroup members would then also increase the average association rates, with little difference among group-members as predicted by chance [73]. If, however, spatio-temporal associations are distinctively influenced by the presence and/or absence of others [12,70–72], then differential avoidance or attraction towards particular individuals should generate variation in association rates, with individuals co-occurring disproportionately more or less than a random expectation [64,74]. The effect of resource availability on subgroup-size should cause individuals to increasingly associate with less preferred partners as subgroups get larger, favoring a negative relationship between subgroup size and association rate [67]. Patterns of co-occurrence have been repeatedly used to investigate active association processes in animal groups [40,71,73,75], being particularly useful for species where direct interactions are difficult to observe [76], species with high fission-fusion dynamics [77] and where rates of affiliative and agonistic contact-interactions is very low, as occurs with *Ateles* spp. [78,79].

Spider monkeys (*Ateles* spp.) are recognized as high fission-fusion dynamics species [31,34] and have been classified as having a female-dispersing and egalitarian social system [31] based on the socio-ecological model proposed by Sterck et al. [80]. According to this model, groups with poorly defined dominance hierarchies, where females are the dispersing sex, as observed in spider monkeys, should experience scramble competition, with a low occurrence of contests for food within and between groups, owed to an impossibility to monopolize unpredictable and dispersed resources such as ripe fruit [31]. The formation of strong and permanent bonds is considered of low value in this context, particularly among the typically unrelated females [31,80,81]. Thus, changes in fruit availability are expected to exert changes on space-use and social organization as observed by Shimooka [52], with smaller ranging areas and larger subgroups when fruit availability is high and concentrated in clustered patches.

**Fig 1:**
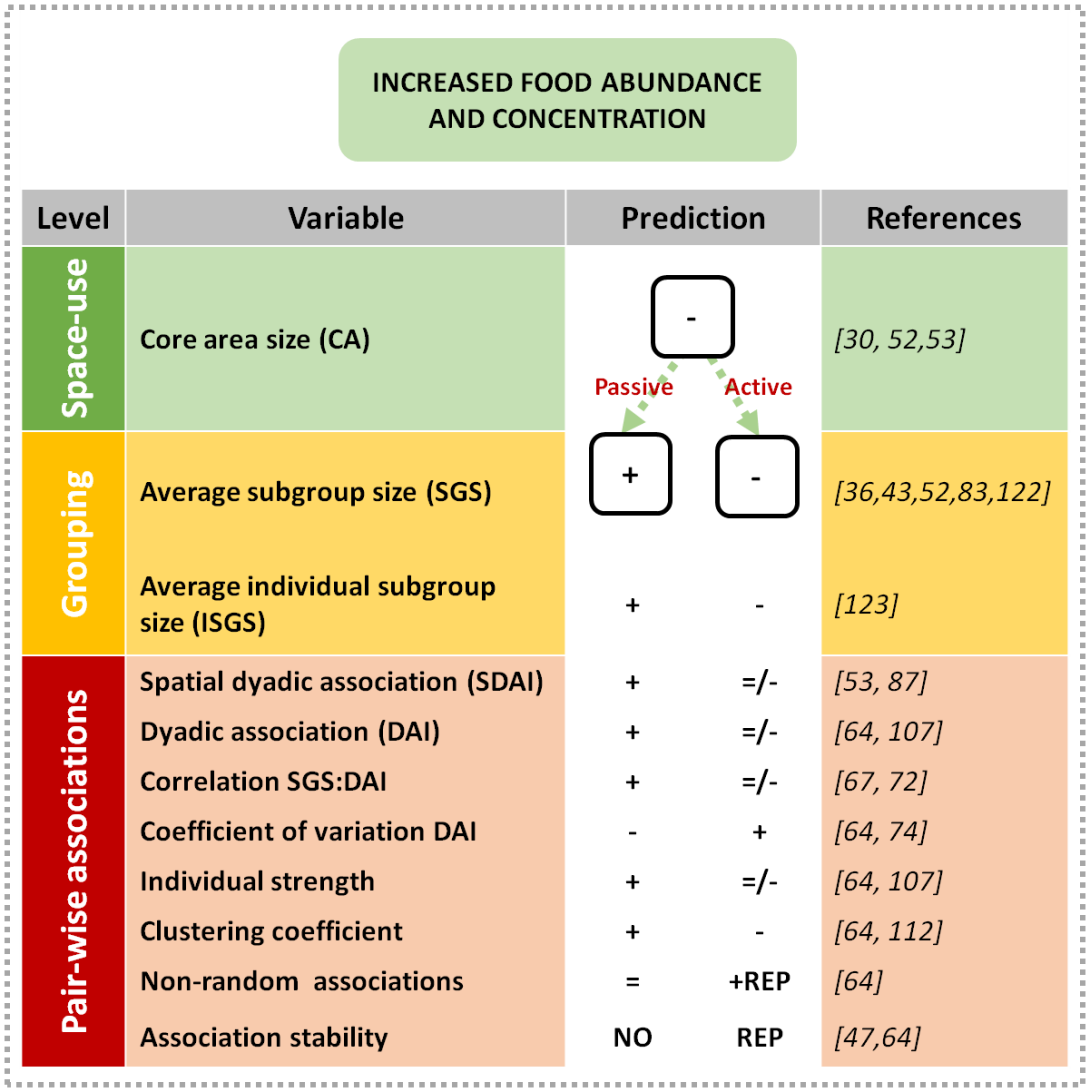
Predicted outcomes in spider monkey association metrics (pair-wise associations) at three levels of analysis for two different socio-spatial contexts (passive *vs*. active) resulting from an increase in food abundance and concentration. Each context is related to either passive or active association processes (legends over dotted arrows). Predictions differ depending on these processes and are expressed as expected increases (**+**), decreases (**-**) or absence of change (**=**). REP indicates that active repulsive associations are expected. Association metrics are described in methods. References indicate papers with theoretical support for the predictions or studies presenting congruent results using the same or equivalent metrics.

The aim of our study was to test whether co-occurrence of individual spider monkeys results from: a) random processes of encounter and aggregation around preferred resources (passive association) or b) individuals actively seeking/avoiding preferred/repelled companions (active associations). To do so, we analyzed temporal patterns in three components of the socio-spatial structure of the group: 1. space-use, 2. grouping tendencies and 3. pair-wise associations. We assumed that an association between any two individuals is not independent of the social context where it occurs (in this case, the size and composition of the subgroup), and that grouping patterns are themselves conditioned to the space being used by individuals (ranging area). Consequently, we formulated a hierarchical-dependence framework for the three components analyzed (Fig 1). We placed space-use at the first level of analysis because it is an indicator of individual spatial decisions which may constrain the likelihood for two individuals to find themselves in the same place at the same time. These decisions may be influenced by individual needs and preferences independent from social factors [46]. In the second level we placed grouping tendencies, which reflect tolerance between individuals and can inform about what brings them together [20]. In social species, subgroup size is expected to increase when food competition decreases [33,43,82]. This response mainly reflects passive association around food patches (which may be enhanced if individuals are also generally attracted to conspecifics), and should be amplified when ranging areas are small because of an increased probability of random encounters among individuals due to higher densities [83,84]. Therefore, deviations from this pattern suggest that active processes of avoidance or attraction could be operating [67]. The first two levels set the context for the third: pair-wise associations between individuals in the same subgroup. The co-occurrence of two particular individuals reflects the effects of the two preceding levels, and may help to further elucidate active and passive processes of association, particularly when analyzed through time [64,85]. This is because variation in the patterns of co-occurrence can inform about the factors that drive two individuals to be together. If individual movement decisions are predominantly influenced by preference for certain companions (active associations), co-occurrence patterns are expected to be relatively stable, despite shifts in ecological conditions, as in the core social tier observed by Wittemyer et al. [41] in African elephants (*Loxodonta africana*). We generated predictions for two combinations of space-use and grouping outcomes, each conforming a socio-spatial context where pair-wise associations could take place. Each of these contexts is related to either passive or active association processes, which were the basis for the corresponding predictions on association variables that represent the third level of the framework, as shown in Fig 1. We analyzed a collection of variables reflecting two types of association: overlap of ranging areas (spatial association) and presence of two individuals in the same subgroup (spatio-temporal association). Each variable from this level captures complementary information about the patterns of co-occurrence used to jointly assess the influence of passive and active processes of association.

Our prediction scheme states that general increases in food abundance and concentration should result in smaller ranging areas (first level of analysis; [30]). By increasing the probability of encounter among individuals [86], larger subgroups would form simply by random aggregation (passive association) facilitated by reduced scramble-competition in the fruit-abundant condition (second level of analysis). If associations among individuals are basically a consequence of these processes, results associated with an increase in food-abundance should follow the prediction for passive associations (third level of analysis; Fig 1). Fruit abundance should allow more individuals to use common patches and therefore ranging areas should overlap more [53,87] increasing spatial dyadic associations. Individuals are therefore expected to associate in subgroups more regularly and with more individuals irrespectively of their identities, increasing the general spatio-temporal association intensity (dyadic associations) and reducing its variability within the group. If individuals increasingly associate with others as a result of co-occurring more often in larger subgroups, this should increase the correlation between subgroup size and spatio-temporal associations. By associating indifferently with more individuals, a general increase in connectivity between all group members is expected in the absence of non-random associations. Accordingly, the framework can be used to establish different scenarios in a set of association variables which depend on individual space-use, spatio-temporal coincidence and the relationship between grouping and association. For example, dissimilar grouping patterns are expected when environmental requirements and motivations differ among group members, as often occurs between sex-classes in many species (e.g. sperm whales, *Physeter macrocephalus*) [68,88].

Sexual differences in space-use and grouping patterns have been well documented in spider monkeys indicating that males are less susceptible to ecological constraints than females [46,52,79,89]. Therefore, female grouping and association patterns should be more dependent on fruit availability (greater influence of passive association processes) than those of males, expected to be relatively stable across seasons (greater influence of active associations). We incorporated these considerations into our general evaluation of individual socio-spatial patterns, by also investigating potential differences between sex-classes using our analysis framework. Consequently, we expected females to follow our predictions for passive association processes as opposed to males, who should show little seasonal variability in their socio-spatial patterns (at all three levels of analysis: cf. Fig 1).

## Methods

### Ethical statement

The present study was conducted in accordance with the guidelines of the Department of Environment and Natural Resources of Mexico (SEMARNAT) under Research Permits DGVS00910/13 and DGVS02716/14. Each permit authorized our research activities with a wild population of spider monkeys (an endangered species) within the *Otoch Ma’ax Yetel Kooh* protected area in Mexico, during 2013 and 2014 respectively. None of the authors had any direct or indirect interaction with the primates in the study.

### Study Site

Field data were collected in the *Otoch Ma’ax Yetel Kooh* protected area in the Yucatan peninsula, Mexico. The 5367 ha area is composed of a mosaic of semi-evergreen forest with different successional stages [90]. Average annual temperature fluctuates around 24°C peaking in August, and 70% of annual rainfall is typically concentrated between the months of May and October [91].

### Study group

The study was conducted on a habituated group of black handed spider monkeys (*Ateles geoffroyi*) ranging around the south-eastern side of the lake located on the eastern margin of the protected area, very close to the village of Punta Laguna [41]. The group has been subject to continuous monitoring since 1997 by local field assistants, researchers and students. Except for infants (age: 0–3 years), group members are all identified through distinctive facial or body marks [92]. Males are the phylopatric sex in this species, while females born in the group usually emigrate at 5–6 years. Before this age, individuals are considered infants or juveniles (age: 3–5 years) and usually move and forage together with their mothers. In this study, we only considered individuals that were five years old by January 2013, additionally excluding group-born females with no offspring, because they mostly followed their mothers, and they all emigrated during the study. This subset comprises all group-members assumed to have independent movement patterns, and will henceforth be referred to as adults. During the study, group size fluctuated between 18 (7 male and 11 female) and 22 (7 male, 15 females) adults due to the integration of four females, two of which had been sporadically observed in the periphery of the group’s home range since 2012. During 2013 three of these females (all with offspring) increased their frequency of visits to the core area of the group, associating regularly with group members and continuing to do so through the rest of the study. All males analyzed were born in the group and both their age and mother are known. Adult female ages and maternity relations are unknown (except for that of a 9 year old natal female (LO) who has reproduced repeatedly in the group) and their tenure in the group varies from 5 to over 18 years for three females which were already part of the group by 1997 when they were first observed.

### Data collection

Throughout a 20 month-period between January 3^rd^ 2013 and September 18^th^ 2014, data were collected by four field assistants (each with over ten years of experience) and one of the authors (SESA), following the same standard methodology implemented at the study site since 1998 [92]. We conducted observations during subgroup follows of 4–8 daily hours which began after opportunistically locating monkeys by searching commonly used areas within the home range. Once a subgroup was spotted, we began our instantaneous scan observations every 20-min registering its composition, position, and the identity and activity of all visible individuals. We obtained the subgroup position using a hand-held global positioning system placed roughly below one of the individuals of the subgroup (mean error in locations was 7 m). Any monkey within 30 meters of another was considered part of the same subgroup [20,93]. This cutoff distance is based on analyses of the distribution of inter-individual distances and has been validated using empirical [93] and modeling [20] methods. When individuals joined a subgroup, we recorded a fusion if they remained present in the following scan. When individuals were out of sight for two consecutive scans, we recorded this event as a fission. Our data comprise 4916 subgroup scans collected during 325 days within four seasons: dry 2013, wet 2013, dry 2014 and wet 2014. For all our analyses, we defined seasons as beginning in November 15^th^ (dry) and May 15^th^ (wet).

### Fruit availability

For a general estimate of fruit availability within the study site, we used an index of fruit abundance [45] calculated using data from a phenological trail monitored fortnightly, recording the presence or absence of fruit in 10 individual trees of each of 11 tree species most consumed by the study group over the long-term [94]. We also used tree-density estimations for these species, and measures of the diameter at breast height (DBH) obtained from trees (larger than 10 cm DBH) in forty-eight 100 x 2 m^2^ linear transects and four square transects of 0.25 ha in the study site [95]. The index of fruit abundance is the resulting sum of the proportion of trees with fruit (out of 10) from each species per fortnight, multiplied by the density (individuals/ha) and the sum of the DBH per ha for each species [45]. An example calculation for species *Sideroxylon foetidissimum* in one fortnight would be: 8/10 fruiting trees * 12 trees/ha * 150cm/ha = 1440 (trees*cm)/ha^2^. This was done for each species with fruit during a given fortnight, and the resulting numbers were added to obtain the index for that particular period. Values of the index showed a significant increase in fruit abundance during wet *vs*. dry months (ANOVA: df = 3, *F* = 17.7, *P*<0.0001; *post hoc* Tukey’s HSD: dry *vs*. wet 2013 *P* = 0.001, dry *vs*. wet 2014 *P*<0.0001) with no yearly differences between seasons and no differences between years (S1 Fig). This pattern largely reflects the abundance of *Brosimum alicastrum*; a species worth highlighting for its relevance in the diet of the study group [45,96] and high density in the study area [95]. The study group has been shown to consistently concentrate its long-term activities in a core area that contains a greater proportion of mature forest than the protected area as a whole (more than 50% *vs*. less than 25% [41]). Considering that *B*. *alicastrum* is ten times as abundant in the mature forest than in other successional stages, fruiting of this species during the wet season results in large and abundant patches within the area where the monkeys concentrate their activities [41]. Therefore, the change from dry to wet season is accompanied by a combination of increased fruit abundance and density of patches within the ranging area of the study group.

### Data analysis

Since the decision to follow a subgroup each field day was not based on the membership of any particular individual, differences in individual spatial preferences produced uneven sample sizes across individuals. To reduce the resulting bias, we restricted our data set to adult individuals observed throughout the four seasons of the study in at least 10% of the total scans from each period. Consequently, all our analyses used data on 11 adult monkeys (four males and seven females; S1 Table) with the exception of the analysis of grouping patterns which included data for any of the adult individuals considered as part of the group each season. Our sample of 11 individuals represented 61% of adult group-members in 2013 and 50% in 2014. This number of focal individuals is comparable to other studies of similar length which analyzed individual ranging patterns in *Ateles* spp. in the same and other study sites (for example: 6 adults and subadults [89], 13 adults [97] and 11 independently moving individuals [49]).

#### Space-use (analysis level 1)

Seasonal ranging patterns were evaluated by individual core areas, which indicate the area where individuals concentrated their activities in parts of the habitat assumed to hold important resources for them [41,51,98,99]. Core areas were defined as the portion of the utilization distribution contained in the 60% probability contour, as calculated by the Local Convex Hull (LoCoH) method [100,101]. LoCoH is a non-parametric method for calculating utilization distributions based on the construction of polygons (hulls) around each observation point using *n* nearest neighbors. The union of these hulls renders areas containing different proportions of points that can be associated with probabilities of occurrence. We used the same definition of core area as Ramos-Fernandez et al. [41], who analyzed ranging patterns for the same group, also using subgroup scan data. From the area *vs*. probability curve for yearly subgroup utilization distributions, they found that a 60% probability best approximated a slope of 1 for all cases. This is indicative of the greatest difference between the empirical curve and the null expectation of random use with no activity clumping [102]. Seasonal core areas were generated for each individual using all scan locations where it was observed. All core areas were calculated using the R software platform (v. 3.1.2 [103]) and the adaptive mode version of T-LoCoH [101]. In this setting, the T-LoCoH adaptive mode parameter *a*, is an upper bound on the sum of distances from each point to neighbors progressively further from it, thereby resulting in variation in the number of neighbors *n* used in the construction of each hull (viz: points in dense clusters have a larger *n* than points that are more isolated from their neighbors). The *a* value was selected through a compromise between minimizing the number of separate patches conforming the utilization distributions and avoiding polygons from crossing natural barriers into areas known not to be used by the monkeys, such as the lake (S2 Fig). The same *a* value was used for all calculations of seasonal-individual core areas.

In addition to individual core area size, we examined seasonal changes in the spatial coincidence of core areas by first quantifying the total area covered by the union of all individual core areas per season, and then identifying the number of overlapping core areas within each portion of this union. We also used two indices to quantify the general coincidence between individual core areas: a group spatial gregariousness index quantifying how clumped together were individual core areas with respect to the total extent covered by the union of all core areas, and the spatial gregariousness of each individual quantifying how much each core area coincided with the rest of the core areas. Both indices are adapted from the index used by José-Domínguez et al. [104] to quantify site fidelity, but instead of considering the overlap of core areas from different time periods, we used the overlap of core areas from different individuals. Group spatial gregariousness was defined by:

$$\text{gSGI}=\left(\Sigma_{i=2}^{j}i*O_{i}\right)/\left(K*A\right),$$

where *A* is the total area covered by the core area union; *j* is the maximum number of overlapping individual core areas in a certain season (11 in all cases); *i* is the number of overlapping core areas with values between 2 and *j*; *O* is the size of the area where *i* core areas overlap within the core area union; and *K* is the total number of core areas analyzed per season (11 in all cases). Values of the group spatial gregariousness index range between 0 and 1 where 1 indicates total spatial overlap of all possible core areas and 0 indicates no coincidence at all (i.e. completely non-overlapping core areas). To calculate the individual spatial gregariousness for individual *x*, we used a very similar formulation where instead of *A*, the denominator includes the individual’s core area *A*_*x*_, and the overlap *O*_*i*_ is restricted to areas of overlap within *A*_*x*_, becoming *O*_*ix*_ in the expression ${\text{iSGI}}_{x}=\left(\Sigma_{i=2}^{j}i*O_{ix}\right)/\left(K*A_{x}\right)$. Values of this index also range between 0 and 1 where 1 indicates total spatial overlap of the individual’s core area with all other possible core areas and 0 indicates no coincidence at all between that individual’s core area and any other (S3 Fig).

#### Grouping tendencies (analysis level 2)

Grouping patterns were analyzed using average seasonal subgroup size (subgroup size) and the average subgroup size experienced by each individual (individual subgroup size). For calculating subgroup size, we included all subgroup scans where at least one of the adult monkeys of the group was in sight, and counts only included adult individuals. Therefore, subgroup size can have values higher than 11 since it considers all adults present at the time. Individual subgroup size was calculated for each individual, by including only scan points where it was in sight. In combination with the predicted decrease in core areas, both subgroup metrics were expected to increase in the food-abundant season if individuals were mostly driven by passive aggregation. Individual subgroup size was additionally used to detect potential differences in the effect of passive and active processes of association on individual gregariousness [74].

#### Pair-wise associations (analysis level 3)

Our analyses of associations were based on two indices, each constructed from a different type of co-occurrence between pairs of individuals: spatial dyadic association index and dyadic association index. Both indices are based on the simple ratio dyadic association index [105,106] which describes the association between individuals A and B by:

$$\text{Association }\left(\text{A},\text{B}\right)\;=N_{\text{AB}}/(N_{\text{A}}+N_{\text{B}}-N_{\text{AB}}),$$

where *N*_AB_ corresponds to the number of co-occurrences of individuals A and B, while *N*_A_ refers to all the occurrences of A and *N*_B_ to all the occurrences of B. Given our sampling methodology, this index is also equivalent to the twice-weight association index [105].

The spatial dyadic association index examined the overlap between the core areas of pairs of individuals, capturing the extent to which they concentrated their activities in the same places during a certain season (spatial association), irrespectively of whether or not they were also observed in the same subgroup. Thus, a co-occurrence between individuals A and B (*N*_AB_ in the association formula) corresponds to the size of the overlap of their core areas, while the occurrence of each individual *N*_A_ and *N*_B_ is the size of each of their individual core areas. Core area overlaps were computed using T-LoCoH. Values of the spatial association index reflect the overlap between core areas of two individuals as a proportion of the total area covered by both core areas.

The dyadic association index describes spatio-temporal associations, where a co-occurrence of A with B refers to the presence of both individuals in the same subgroup (*N*_AB_ in the association formula). This necessarily involves that they were together at the same time, and therefore captures the dyad’s tendency to occur in the same subgroup (dyadic association). Variables *N*_A_ and *N*_B_ correspond to all the observations of A or B in a subgroup. Average seasonal dyadic association index therefore increases if the number of associates of individuals and/or the intensity of their associations increase [64,107].

Changes in the size and location of the area used by individuals can modify the probability of random encounter with others. Variation in this random probability of encounter compared to variation in real encounter rates between pairs of individuals can indicate the influence of random processes of aggregation in patterns of association. To evaluate if any observed changes in core areas affected the probability of encounter, we ran a Monte Carlo simulation using T-LoCoH. For each season and pair of individuals, we assumed a random uniform distribution within each of their core areas. The simulation consisted of independent throws where we randomly added a point within the seasonal core area of each individual of the pair. Each pair of points added (one for each individual) was considered a throw. A trial was conformed of *z* number of throws corresponding to the smaller number of observations on the two members of a pair for a given season, because that was the maximum number of times they could have been observed together. For every throw, we measured the distance between the two points and if it was 30 meters or less, the pair was considered to be associated (spatio-temporal co-occurrence) in accordance with our field definition of subgroup (see above). If the distance was greater than 30m, the throw counted as an occurrence of one of the two individuals in absence of the other. We assigned these occurrences to one of the two individuals, alternating them each throw (because only one monkey could be observed at a time with our field methodology). We ran a thousand trials for each pair of individuals per season, averaging the total number of co-occurrences per trial to obtain the average random occurrence for each dyad. We used this value to calculate a random dyadic association index for each pair of individuals, in the same manner as the dyadic association index, but using the average number of random occurrences as the value for the co-occurrence *N*_AB_ (in the association formula), while *N*_A_+*N*_B_ corresponded to *z*. This random association measure is an approximation to the random probability of encounter between individuals, exclusively as a result of the relevance of core area overlap. If core areas decrease in areas commonly used by both members of a dyad, random associations are expected to increase. This random association index was then compared to the dyadic association index based on the observed encounter rates. However, because the random index was restricted to core areas, and the dyadic association index captures processes occurring beyond core areas, we calculated an equivalent of the dyadic association index that only considered occurrences of individuals within their respective core areas. By doing this, we eliminated possible random spatial effects operating outside core areas, potentially contained in the dyadic association index.

Active processes of association can be identified by examining if certain individuals co-occurred more than a random expectation based on each individual’s tendency to associate in general [73]. While the Monte Carlo simulation allowed us to estimate the probability for two individuals to randomly find each other, this did not inform us if the associations observed were any different than expected if individuals chose group partners at random. Bejder et al. [108] devised a method where random sets of data are generated from the original, preserving the number of subgroups in which each individual was observed and the number of individuals in each subgroup. When a large number of random samples are generated, they may be used to distinguish non-random processes in the original data [74]. We ran permutation tests on the compiled version of SOCPROG 2.5 for each seasonal dataset, taking the coefficient of variation of the association index as our test statistic [73,109]. All tests were done using the dyadic association index corrected for gregariousness [110]. This correction accounts for individuals that might prefer certain group-sizes rather than particular companions and is represented by:

$$\text{DAIG}\left(\text{A},\text{B}\right)=\left(DAI_{\text{AB}}\right)\left(\Sigma DAI/\left(\Sigma DAI_{\text{A}}\Sigma DAI_{\text{B}}\right)\right),$$

where *DAI*_AB_ is the dyadic association index between individuals A and B, Σ*DAI* is the sum of the dyadic association index for all dyads observed in a season and Σ*DAI*_A_ and Σ*DAI*_B_ represent the sums of all the dyadic associations for individuals A and B, respectively [110]. As a result, the analysis indicated the occurrence of associations which were stronger (attractive) or weaker (repulsive) than the random expectation based on a predefined significance level (*P* < 0.05 for all tests). Additionally, the test identified non-random dyads, and this subset was used to assess association stability by examining the number of seasons in which each of these dyads was observed. We considered both consecutive and non-consecutive recurrences of non-random associations, because the first inform about the endurance of an association despite the effects of seasonal changes in the socio-spatial context, while non-consecutive associations could reveal driving factors for a particular association in a certain seasonal context. Altogether, this analysis provides criteria to determine the presence and persistence of active processes of association.

A complementary source of insight about the factors influencing observed associations is the social context where they occur, which was not accounted for in previous analyses. We searched for changes in the correlation between the dyadic association index and the average subgroup size, as indicators of the type of association process occurring in each season. Newton-Fisher [67] used this correlation to discern between processes of passive and active association in a group. In the former, dyadic associations are expected to correlate positively with subgroup size, whereas in the latter, higher dyadic association values are expected among individuals that tend to be together in smaller subgroups and therefore the correlation between dyadic associations and subgroup size should be negative. Following methods by Newton-Fisher [67] and Wakefield [72], we examined this correlation by first converting each set of seasonal dyadic association values into a z-score so that they varied on the same relative scale, and facilitate comparison between seasons. We calculated the average subgroup-size for each dyad, and log normalized both variables (previously adding 1 to each dyadic association z-score to make all values positive). Finally, we calculated Kendall’s tau coefficient for each season. If smaller subgroups include individuals with stronger associations [67], differences in association strength should be most apparent in single-pair groups. If this were the case, 1) some dyads should occur in single-pairs relatively more than others and 2) there should be a higher probability of finding attractive associations among those dyads that associate most frequently in single-pairs. To test this assumption we used the results from the permutation tests for non-random associations and a dyadic association index restricted to pairs (pair index), to investigate if dyads with attractive associations were more prone to occur in pairs than others. We calculated the pair index in the same manner as the dyadic association index but taking a subset of the scan-data corresponding only to subgroups of two individuals. For the pair index, the co-occurrence value *N*_AB_ involved both individuals being together in single-pair subgroups and was restricted to all instances where one individual (A) or the other (B) were in a subgroup of size two. We used Mann-Whitney U tests to compare pair index values among dyads with attractive associations against all other dyads.

As a way to quantify association homogeneity and evaluate how it changed between seasons, we calculated the seasonal coefficient of variation (standard deviation relative to the mean) of the dyadic association index using dyadic association values for all dyads from each season [64]. Lower values indicate little difference between dyads in their associations, suggesting passive aggregation processes, while higher values are expected when there are different patterns of association within the group, indicating active processes.

We complemented our analysis of associations with a quantitative exploration of changes in the seasonal association network for the 11 study subjects. We used SOCPROG 2.5 to construct weighted non-directional networks for each season. Nodes represented individuals and weighted links represented the dyadic association index corrected for gregariousness [110]. We used the seasonal change in average individual strength and clustering coefficient of each network to evaluate the stability of the associations through time, which can be indicative of long-term processes of active association [64]. The individual strength corresponds to the added weights of all links connected to a node. It is equivalent to the degree for networks with weights and is a measure of how connected a node is to the rest of the network [74,111]. An increase in the number of associations or their intensity will therefore result in increased individual strength. The clustering coefficient indicates how well the associates of an individual are connected among themselves [112]. The version of the coefficient implemented in SOCPROG 2.5 is based on the matrix definition for weighted networks by Holme et al. [113], where the clustering coefficient of individual *i* is given by:

$${\text{C}}_{w}\left(i\right)=(\Sigma_{jk}w_{ij}w_{jk}w_{ki})/({\text{max}}_{ij}\left(w_{ij}\right)\Sigma_{jk}w_{ij}w_{ki}),$$

Where *w*_*ij*_, *w*_*jk*_ and *w*_*ki*_ are the values of the association indices between individual *i* and all its pairs of associated *jk*, while max_*ij*_(*w*_*ij*_) is the maximum value of the association index of *i* with any individual *j*. As with the dyadic association index, this metric is expected to be higher if individuals increase the frequency of occurrence with their associates from the previous season (*i*.*e*. if they are more strongly connected), or if they increase the number of individuals with which they occur (*i*.*e*. if individuals are connected to an increased number of others).

#### Statistical analyses

Seasonal comparisons were done using Wilcoxon signed-rank tests unless specified otherwise. For other comparisons we used Mann-Whitney U and Kruskal-Wallis tests. *P* values for pair-wise differences after multiple comparisons were adjusted with the Bonferroni correction (*P*_adj_). When presented, bootstrap confidence intervals were obtained by resampling the corresponding original data 1000 times with replacement. A distribution of averages was then used to derive 95% confidence intervals using the first-order normal approximation as implemented in the boot package for R [114].

## Results

### Space-use

**Fig 2:**
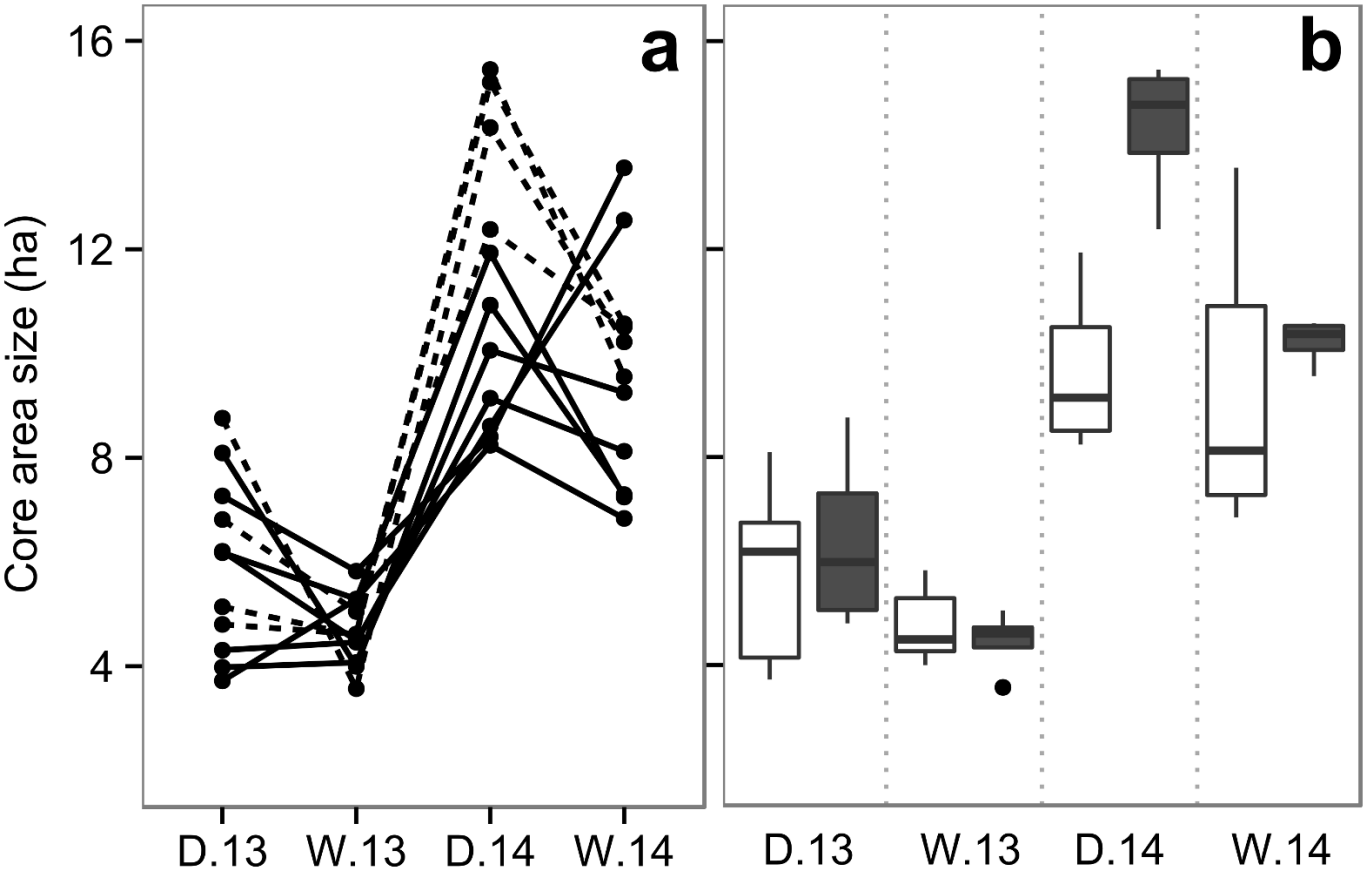
**(a)** Seasonal change in individual core area size for the females (solid lines) and males (dashed lines) of the study group. **(b)** Grouped differences between females (white) and males (gray). The point represents an observation outside 1.5 times the interquartile range above the upper quartile and below the lower quartile.

Seasonal individual core areas ranged in size between 3.57 ha and 15.45 ha, with an average of 7.88 ha (±3.57; S2 Table). Although core areas were smaller in wet *vs*. dry seasons (*W* = 205, *n* = 11, *P*<0.01), within years, the seasonal change was only significant for the dry *vs*. wet season of 2013 (*W* = 56, *n* = 11, *P* = 0.04) and not for the dry *vs*. wet season of 2014 (*W* = 50, *n* = 11, *P* = 0.1). The most salient difference, however, was between years, with core areas being larger during 2014 (*W* = 253, *n* = 22, *P*<0.0001; Fig 2a). When comparing between sex classes, differences were only significant in the dry season of 2014 when males had larger core areas than females (Mann-Whitney: *U* = 28, *n*_males/females_ = 4/7, *P*<0.01; Fig 2b). So, as predicted (Fig 1), the change from fruit-scarce to fruit-abundant seasons was accompanied by a general contraction of individual core areas although less so in 2014 and with greater difference between sexes than in 2013.

The spatial overlap of core areas indicated an expansion of the total extent covered by all individual core areas (core area union) during dry *vs*. wet seasons, but mostly in 2014 *vs*. 2013 (S2 Fig). Core area union was largest in the dry season of 2014 (24.5 ha) and smallest in the wet season of 2013 (12.4 ha), while the 11 core area overlap varied in size between 1.8 ha (wet 2014) and 0.7 ha (wet 2013; Table 1, S4 Fig). We used the group spatial gregariousness index to quantify the general degree of core area overlap, finding it was similar for all seasons, fluctuating between 0.50 and 0.54 (S3 Table). This indicates little change in the proportional spatial clumping of core areas in all periods. Similarly, the individual spatial gregariousness index showed no significant differences between seasons or years, but average individual values of the index were significantly higher for females than males (Mann-Whitney, *U* = 28, *n*_males/females_ = 4/7, *P*<0.01; S3 Table, S5 Fig). This result indicates that females tended to have a higher core area overlap with the rest of the individuals analyzed (female or male), than any male. We then investigated sexual differences in the core area overlap among individuals of the same sex by calculating the individual spatial gregariousness index by sex. Considering only the core area overlap within sexes, the average values of the index by sex indicated significantly higher spatial coincidence for males than females (Mann-Whitney, *U* = 28, *n*_males/females_ = 4/7, *P*<0.01; S6 Fig)

**Table 1:** Seasonal extents of the union of individual core areas (CA union) and the area of overlap for all 11 core areas (11 CA overlap).

	DRY2013	WET 2013	DRY2014	WET2014
**CA union (ha)**	14.1	12.4	24.5	22.2
**11 CA overlap (ha)**	1.2	0.7	1.8	1.8

### Grouping tendencies

**Fig 3:**
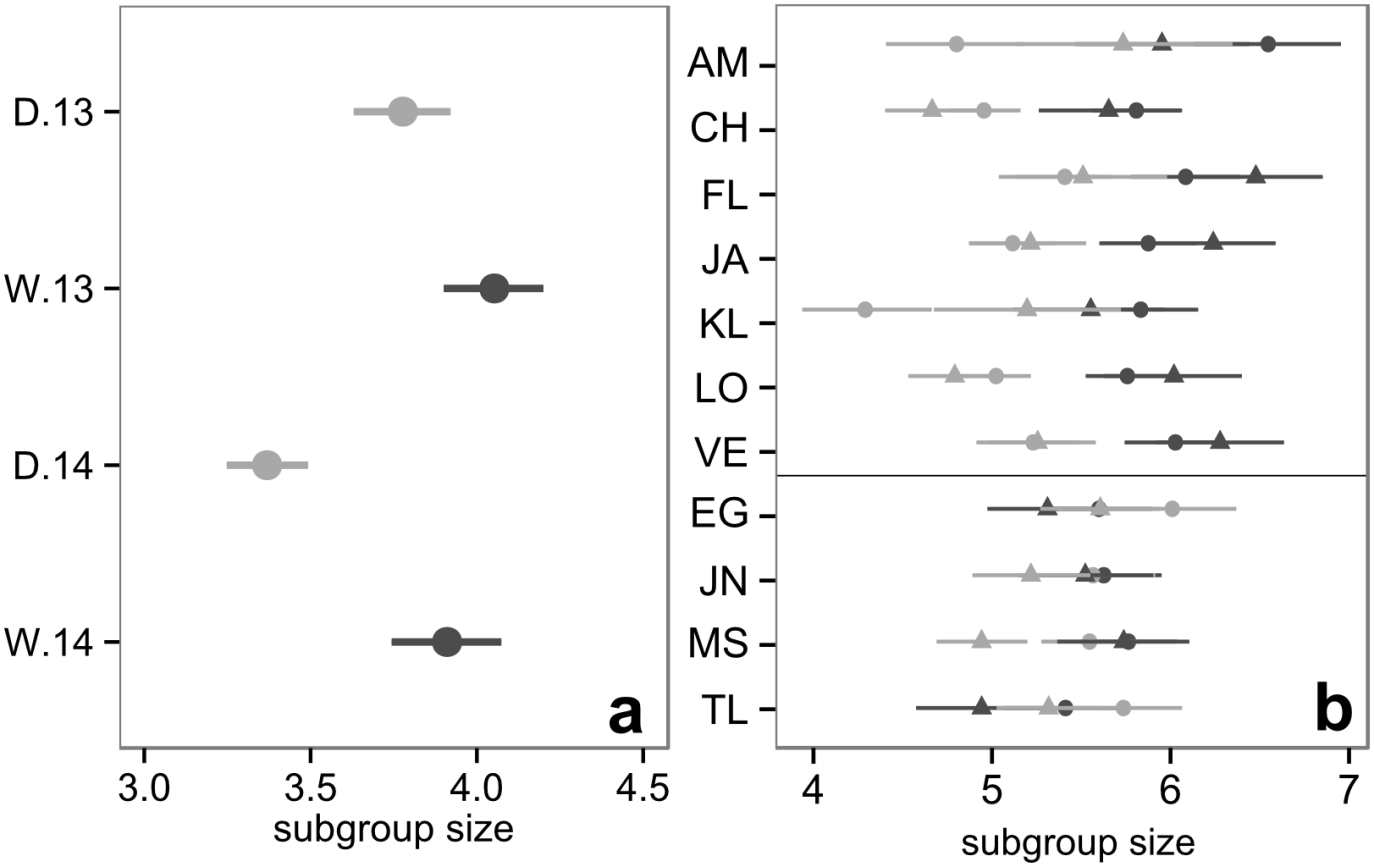
**(a)** Average subgroup size during the dry (light gray) and wet (dark gray) seasons of 2013 and 2014. **(b)** Average subgroup size experienced by each individual during the dry (light gray) and wet (dark gray) seasons of 2013 (circles) and 2014 (triangles). Each row represents an individual identified by a two-letter name code, with females above the black line and males below. Bootstrap confidence intervals (95%) shown in both figures were derived from 1000 replications of the original data (D.13: dry 2013, W.13: wet 2013, D.14: dry 2014 & W.14: wet 2014).

Subgroup size was smaller in dry *vs*. wet seasons (Mann-Whitney, *U* = 3201118, *n*_DRY/WET_ = 2529/2312, *P*<0.0001), although the yearly seasonal increase was only significant in 2014 (Mann-Whitney, 2013: *U* = 649585, *n*_DRY13/WET13_ = 1015/1329, *P* = 0.1; 2014: *U* = 646713.5, *n*_DRY14/WET14_ = 1514/983, *P*<0.0001; Fig 3a). Individual subgroup size increased significantly in both wet seasons (2013: *W* = 7, *n* = 11, *P* = 0.02; 2014: *W* = 7, *n* = 11, *P* = 0.02) suggesting that, the monkeys experienced a higher degree of gregariousness during the wet season, as predicted for passive associations. This change was mostly observed in females (Fig 3b), and two of them (AM and KL) followed the same pattern as the others, but less so during 2014. As expected, male grouping tendencies were more stable across seasons indicating they were less influenced by passive association processes than females. Differences in the size of subgroups of different sexual composition are presented in S4 Table.

### Pair-wise associations

**Fig 4:**
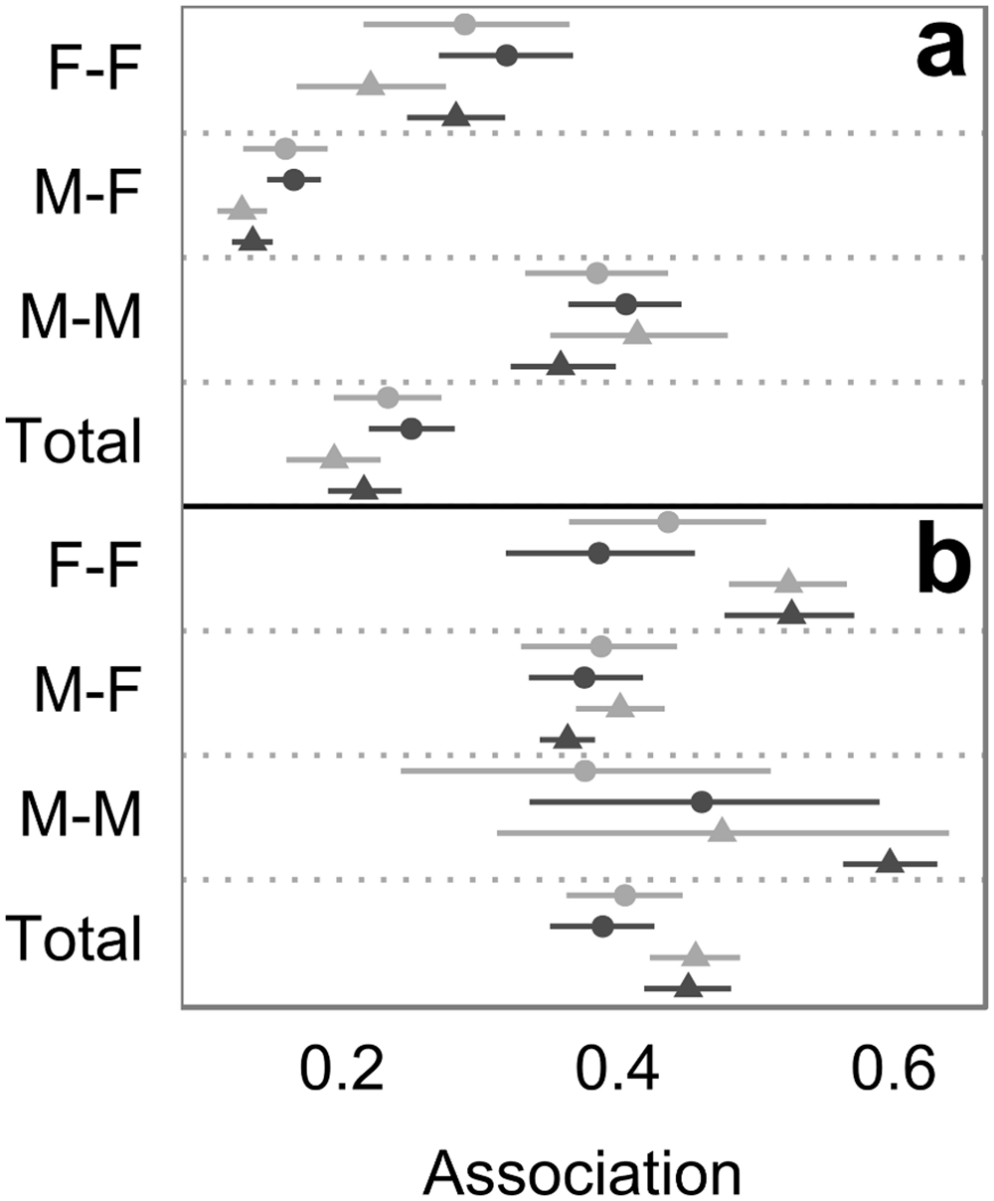
Average seasonal values for (**a**) the dyadic association index and (**b)** the spatial dyadic association index, during the dry (light gray) and wet (dark gray) seasons of 2013 (circles) and 2014 (triangles), grouped by the sexual composition of dyads: female-female (F-F), male-female (M-F), male-male (M-M), and all together (Total). 95% bootstrap confidence intervals were derived from 1000 replications.

As in the case of the subgroup size, the dyadic association index followed the prediction for passive association, with higher values in wet *vs*. dry seasons (*W* = 2282, *n* = 110, *P* = 0.02), but with yearly seasonal differences only significant in 2014 (2013: *W* = 639, *n* = 55, *P* = 0.3; 2014: *W* = 530, *n* = 55, *P* = 0.04). Additionally, we observed higher seasonal dyadic association averages in 2013 *vs*. 2014 (*W* = 4544, *n* = 110, *P*<0.0001; Fig 4a). When considering the sexual composition of the dyad, female-female dyads (F-F) followed the overall dyadic association pattern (2013: *W* = 83, *n* = 21, *P* = 0.2, 2014: *W* = 39, *n* = 21, *P* = 0.006), while seasonal dyadic association values for mixed sex (F-M) and male-male (M-M) dyads were not significantly different in any case. In all seasons, same-sex dyads had significantly higher values of the dyadic association index than M-F with the exception of F-F dyads in the dry season of 2013, which were not significantly different than M-F (S5 Table). In the dry season of 2014, M-M also had significantly higher dyadic association values than F-F (*U* = 13, *n*_FF/MM_ = 21/6, *P*_adj_ = 0.006). As expected, these sexual differences point to sexual segregation, with more stable associations among males than females.

Contrary to prediction under a passive association scenario, the spatial association index showed no significant differences between seasons. This indicates that the proportion of shared core area between dyads did not change seasonally as expected if individuals had increasingly used the same food patches in the food abundant periods. Additionally, we found that spatial associations were significantly lower for M-F than for F-F dyads in the dry and wet seasons of 2014 and for M-M in wet 2014 (Fig 4b; S6 Table). The fact that F-F dyads had higher spatial association values than F-M in both seasons of 2014 indicates that females were sharing areas of use among themselves more than with males, irrespectively of the season (S7 Fig).

The random association index showed a significant increase in the wet *vs*. dry season of 2013 (*W* = 430, *n* = 55, *P*<0.01), but no change between seasons in 2014 (*W* = 612, *n* = 55, *P* = 0.2), indicating that individuals were significantly more prone to find another by chance in wet *vs*. dry 2013, while in 2014 there were no seasonal differences in this respect. Meanwhile, dyadic associations within the core areas did not show seasonal changes (2013: *W* = 559, *n* = 55, *P* = 0.08; 2014: *W* = 552, *n* = 55, *P* = 0.07; S8 Fig). Therefore, this result did not reflect the seasonal increase in the probability of random encounter in 2013 as would be expected if co-occurrence was mostly prompted by this process in a passive association scenario. Similarly, the lack of seasonal change in the random association index in 2014 makes it unlikely that the seasonal increase in dyadic associations was related to this spatial effect.

Permutation tests highlighted associations that occurred both more (attractive) and less (repulsive) than the random expectation in the four seasons analyzed, detecting a maximum of 11 in the wet season of 2013 and a minimum of 4 in the dry season of the same year, for a total of 32 (S7 Table). All the seasonal results were above the expected number of non-random associations by chance (2.75). Of all the significant associations expected, only one dyad was present in all four periods with an attractive-type of association. This is the only dyad conformed by a female and her adult daughter (CH and LO). Since dyadic association values for this dyad were always the highest in each season, and mother-daughter pairs are uncommon in spider monkey groups given that subadult females usually migrate, we ran a second permutation test removing LO (the adult daughter of CH) from the analysis. This allowed us to detect additional non-random associations, previously undistinguished due to the outlying values of the dyadic association index between CH and LO, particularly during 2013 (S7 Table). Most associations identified in the first test also resulted non-random in the second run, with the exception of one repulsive in the wet season of 2013 (JA-MS) and three attractive associations in wet 2013 (EG-TL), dry 2014 (MS-TL) and wet 2014 (FL-JA), respectively. Combining both tests (with and without LO), we detected a maximum of 13 of these associations in the wet season of 2013, and a minimum of 7 in the dry season of 2013 (S7 Table; S9 Fig) for a total of 38 overall. Results include dyads with associations in two consecutive seasons (5 in total, 3 attractive and 2 repulsive), in non-consecutive seasons (1 attractive and 1 repulsive) and 1 dyad with an attractive association in one season and repulsive in another. The latter involved JN, the only male that had attractive associations with any female (three in total) and only in the dry season of 2013. Besides these cases, all non-random male-female associations were repulsive, and all attractive associations occurred among same-sex dyads (S10 Fig).

**Fig 5:**
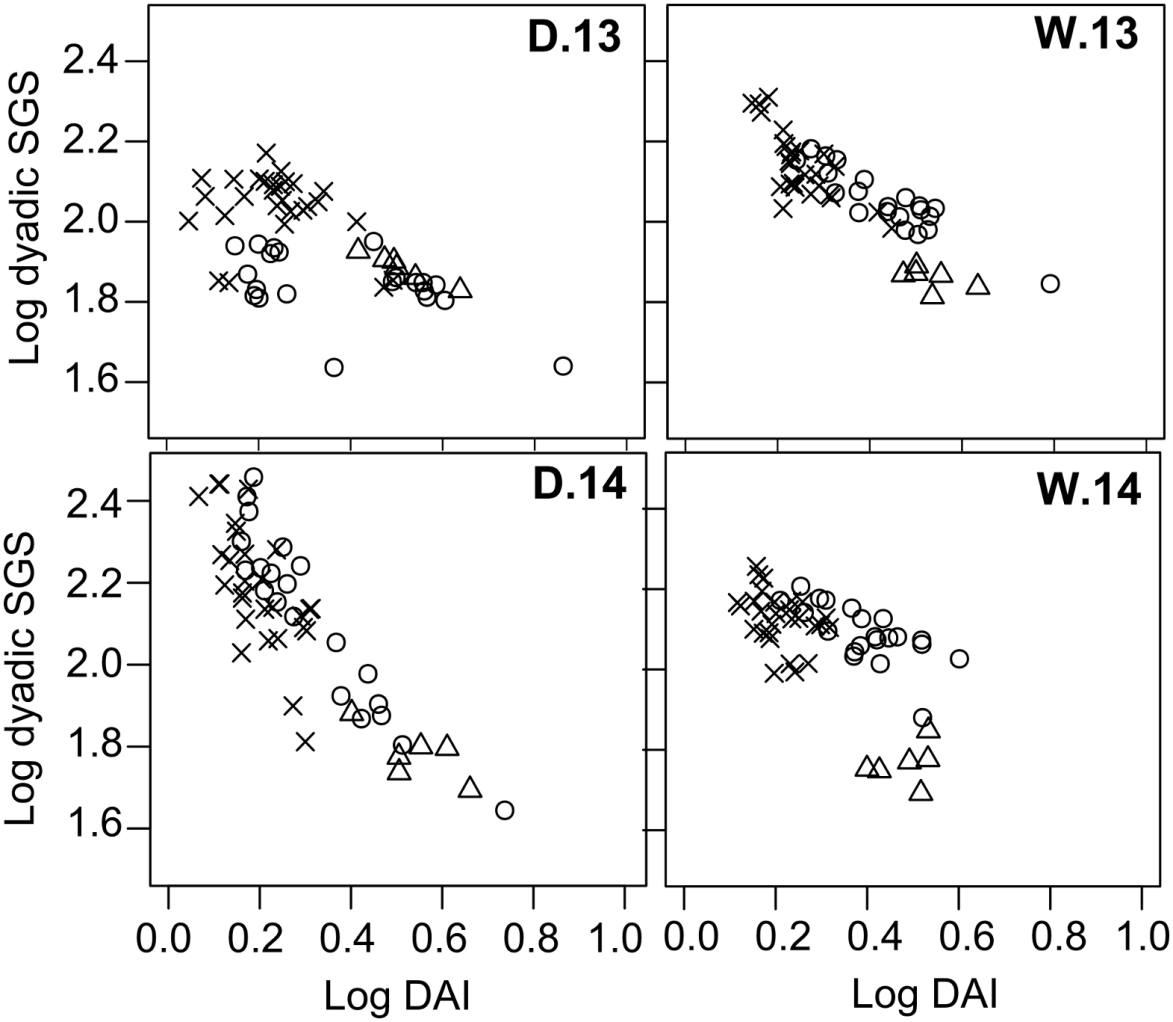
Average dyadic subgroup-size (SGS) as a function of the dyadic association index (DAI) during the dry (left column) and wet (right column) seasons of 2013 (top row) and 2014 (bottom row). Each point corresponds to a female-female (circles), male-male (crosses) or male-female (triangles) dyad.

Correlation values between the dyadic association index and the average subgroup size for each dyad were negative in all four seasons analyzed, showing that dyads associating in smaller subgroups tended to have stronger associations (Fig 5). This is indicative of an active association process under the assumption that, as subgroups split and get smaller, individuals remain with associates they prefer or at least are not repelled by. This assumption was supported by differences in the dyadic association index restricted to pairs, which was significantly higher for dyads with attractive non-random associations (Mann-Whitney: *U* = 3413, *n*_att/non.att_ = 22/198, *P*<0.0001) than for the rest. This was also the case for each season individually, except for the dry season of 2013 when there were no significant differences between attractive associations and the rest. Therefore, dyads that associated more than expected by chance, according to the permutation tests, also tended to occur in single-pair subgroups more than the other dyads. When looking at seasonal differences we found that the correlation between subgroup size and dyadic associations went from a value of Kendall’s correlation coefficient, *K*_*τ*_ *=* -0.36 in dry 2013 to *K*_*τ*_ *=* -0.66 in wet 2013 and from *K*_*τ*_ = -0.64 in dry 2014 to *K*_*τ*_ = -0.44 in wet 2014 (*n* = 55, *P*<0.0001 in all cases). According to our predictions, the shifts in the correlation suggests that in 2013 there was an increased effect of active associations in wet *vs*. dry 2013 while in 2014 the pattern supports the hypothesis of an increased effect of passive associations for the wet with respect to the dry season of 2014.

We used the coefficient of variation of the dyadic association index as an indicator of the homogeneity of associations. Our results showed decreases in both wet seasons with respect to dry seasons (dry 2013: 0.64, wet 2013: 0.49, dry 2014: 0.65, wet 2014: 0.49) with no observed differences between years, indicating that associations were more homogeneous in the food-abundant periods. This supports the prediction for passive associations because individuals appear less selective of their associations in the fruit-abundant periods, as expected if they were mostly co-occurring around resources of common interest.

Changes in individual strength in the association networks were used as an indication of the stability of individual’s tendency to associate with others. Average individual strength had its highest value in the wet season of 2014, after a significant increase with respect to dry 2014 (*W* = 1, *n* = 11, *P* = 0.002), while there were no differences between seasons in 2013 (*W* = 44, *n* = 11, *P* = 0.3; S7 Table). The results for 2014 indicate that individuals tended to have stronger associations with others in the wet season, as predicted for passive associations when individuals can aggregate in larger subgroups and for longer periods if resources are abundant enough. Conversely, the lack of change in average strength in 2013, points to active association processes.

By looking at the clustering coefficient, we measured how connected individuals tended to be with the rest of the network. The clustering coefficient of the association networks increased significantly in both wet seasons with respect to the preceding dry periods (2013: *W* = 66, *n* = 11, *P* = 0.003; 2014: *W* = 66, *n* = 11, *P* = 0.003; S7 Table) as predicted for the passive association hypothesis.

**Fig 6:**
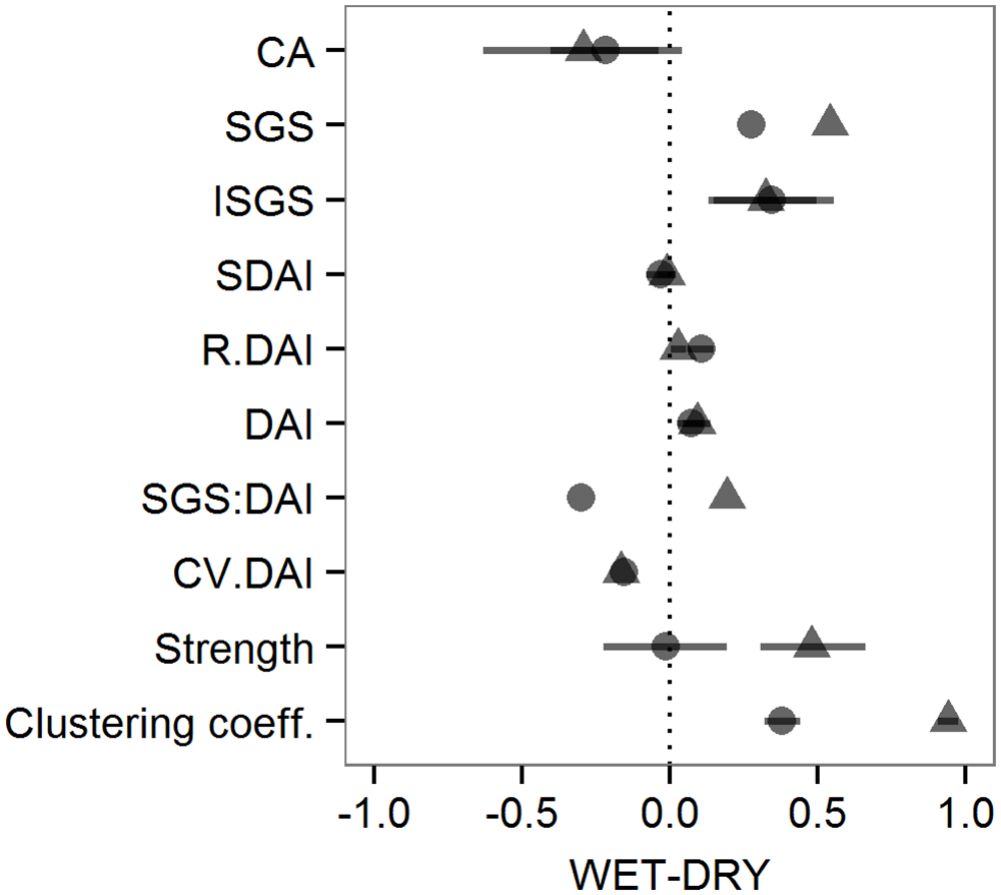
Seasonal change in socio-spatial variables (y-axis) in the wet *vs*. dry seasons of 2013 (circles) and 2014 (triangles). Results are presented as normalized differences between dry and wet seasons. Positive values indicate increases from the dry to wet season, negative values are decreases and values at zero indicate no seasonal change. 95% bootstrap confidence intervals were derived from 1000 replications of the seasonal differences in each variable (CA: core area; ISGS: individual subgroup size; SDAI: spatial dyadic association index; R.DAI: random dyadic association index; DAI: dyadic association index; Strength: individual network strength; Clust Coeff: clustering coefficient), excepting the average subgroup size (SGS), the coefficient of variation for the dyadic association index (CV.DAI) and the correlation between subgroup size and dyadic association index (SGS:DAI). Variables correspond to those presented in the 3-level analysis framework (Fig 1), also including the random probability of encounter measured through R.DAI.

Fig 6 is a visual summary of the seasonal differences that we found in the variables as we predicted in our framework (Fig 1). Overall, space-use and individual gregariousness were supportive of the passive association hypothesis as observed in the seasonal decrease in core area, and the increase in individual subgroup size. Following the 3-level analysis framework for a socio-spatial context driven by passive associations (Fig 1), both wet seasons resulted in significant increases in clustering coefficient values, and decreases in the coefficient of variation for the dyadic association index. However, spatial association values did not change in either year, contrary to the expectation for this context. Moreover, the seasonal pattern in the correlation between subgroup size and dyadic associations changed in opposite directions each year, decreasing in 2013 and increasing in 2014. Only the latter agreed with the prediction for the corresponding socio-spatial context. Similarly, the patterns for subgroup size, dyadic association index and individual strength only partially followed the expected outcome, increasing significantly in 2014 but not in 2013. The latter results are suggestive of active avoidance processes operating in 2013, particularly considering the seasonal increase in the random association index (derived from core area contraction with equivalent spatial coincidence) that would have facilitated the encounter of individuals and the formation of larger subgroups during the wet season. On the other hand, the seasonal increase in subgroup size in 2014 corresponds to the expectation for passive associations but with little influence from the spatial context, given that neither core area nor the random association index showed seasonal changes. Altogether, our results show yearly differences in the socio-spatial context, which agree with a seasonal increase in the influence of passive associations during both wet seasons, but also provide evidence of active associations in all seasons, particularly pointing to active avoidance as a constraint on grouping patterns in 2013.

## Discussion

Our results are indicative of an increased effect of passive processes of association during fruit-abundant periods. We also found evidence of active associations (both repulsive and attractive) in all the periods analyzed, although unstable across seasons. As predicted, a more concentrated use of space in the fruit-abundant periods was related to individuals forming larger subgroups, which in turn led to higher association rates with less variation among dyads. At the same time, results point to yearly differences in the socio-spatial context, apparently not driven by ecological differences. This annual variation was reflected in most association variables, possibly revealing the influence of active associations on the grouping decisions of individuals through avoidance.

Both wet seasons presented evidence that individuals occurred in larger subgroups, pointing to a scenario with prevailing processes of passive association. This was further supported by results in some of the association variables as expected if individuals coincided more often at food sources irrespectively of their identities, and patches could sustain a greater number of individuals than in the dry season, allowing them to remain in larger aggregations. These results are coherent with observations from other groups of *Ateles* spp. where ranging and grouping patterns have corresponded similarly to fruit distribution and availability [43,46,52,53]. In particular, intra-group competition as a constraint on the size of subgroups could be relaxed in times of high food availability and allow the formation of larger subgroups [34,43,115, although see 22]. When combined with a more confined use of space, this pattern suggests that the conformation of subgroups in food-abundant conditions can basically result from individuals prone to aggregate, randomly coming across the same food patches, as proposed by Ramos-Fernández et al. [63]. The gregarious propensity could be a consequence of the history of ecological pressures, such as the distribution of food sources and the risk of predation [31]. While food-availability has been related to group-size in spider monkeys [34,43,115], the extent to which this may be influenced by general attraction towards conspecifics remains unclear [22,23]. Predation risk is considered to be low for spider monkeys [31,43,63], yet frequent observations of subgroups with multiple individuals displaying alarm calls [116, personal observations] and evidence of reduced vigilance related to the number of group members in proximity [117], suggest that this factor should not be disregarded as a possible driver of gregariousness in the species. Furthermore, annual differences revealed by our analysis also suggest that active processes of association can potentially influence grouping decisions and consequently, add support for the relevance of social factors in shaping the grouping behavior of this species [23,79,89].

As we expected, individual core areas tended to contract in the wet season, a condition of increased food abundance and concentration. However, the most salient change was between years: in 2014, core areas were almost twice the size observed in 2013; a difference not clearly related to general fruit abundance patterns for each year. Given the resolution of our ecological data and the known influence of patch distribution (particularly considering species other than *B*. *alicastrum*) on ranging patterns [30,118], we cannot discard an unaccounted effect of food distribution on the yearly differences observed. Yet, the expansion of core areas in 2014 could have also been influenced by the integration of four females into the group during 2013. The fact that two of them had been sporadically observed in the periphery of the group’s home range since 2012, suggests they might have been familiar with the area by 2013. This contrasts with the usual immigration situation wherein females are presumed to come from distant groups [119], implying they would be naïve about the environment [61] and the individuals they encounter [81]. It is also possible that these females had been part of a neighboring group that seemingly moved further away from the study group’s home-range after 2005 [41,120], or had simply settled in an area uncommonly monitored, thereby making encounters with group-members hard to register. In either case, the integration of females familiar with the area into the group could have influenced the general expansion of core areas, with these immigrant females adding their own knowledge about food availability to the group’s pool of information. These immigrant females might have lead the group into areas that they had previously used, particularly during the food-scarce period following the establishment of regular associations in the wet season of 2013. Moreover, the new females could have particularly influenced the ranging patterns of the males in their attempt to both ensure breeding opportunities and deter males from other groups from approaching the expanded number of females [46]. A previous study found a positive association between group core area and the proportion of males in the group [41], but this is opposite to our results, since the number of females increased throughout the study period with no change in the number of adult males. However, our results do indicate that males jointly changed their spatial behavior between the two years: they frequented significantly larger and coinciding portions of the home range than did females, as evidenced in both seasons of 2014 by the increased tendency for males to have larger core areas and to coincide spatially among themselves more than with females. Unfortunately, the lack of data on the new immigrants prior to their integration to the group, added to small sample sizes once they were regularly observed, did not allow for a quantitative analysis of their space-use patterns to compare with those of the individuals studied.

At this time, we are unable to determine what drove the generalized increase in core areas in 2014, but this expansion was an opportunity to examine two different socio-spatial conditions, with the combination of association variables used. In our analysis, average subgroup size, dyadic associations and network strength, increased in the wet season of 2014 as predicted under the influence of passive associations, but the same did not occur in 2013. One possible explanation for this is that individuals reduced the frequency of their associations, even though they tended to increase the average number of their associates. This would indicate that a process of avoidance may be at play, particularly considering the increased random dyadic association index in the wet with respect to the dry season of 2013. In other words, even if individuals were more prone to randomly find a food patch with other individuals in it, and food availability allowed for larger subgroups, average association rates did not increase in the wet season of 2013. This implies that individuals may have avoided or were repelled by others. Our results therefore suggest that, in addition to ecological influences, social factors may pose constraints on the grouping patterns of spider monkeys.

Permutation tests detected non-random associations in all the seasons analyzed, and this was consistent with all-negative values for the correlation between subgroup size and the dyadic association index, suggesting the constant presence of active processes of association. However, the permutation tests also revealed that, with the exception of one mother-offspring pair, active associations were not stable across seasons. This supports the idea that, given the difficulty to monopolize resources, long-term strong associations are unlikely and of little benefit for females unless they are kin-based [80,81]. Nevertheless, the potential relevance of active association processes is not confined to the effects of attraction-based relationships (*e*.*g*. agonistic support; [121]), but also those regarding repulsion or avoidance. For instance, research on another high fission-fusion dynamics species, the chimpanzee, has shown that low-status females occupy lower-quality core areas, have lower site fidelity and incur in higher energetic costs of foraging than high ranking females [50,122]. Moreover, core area quality has been related to reproductive success and female chimpanzees are reported to be more aggressive within their core areas [60,123]. This all suggests that the space-use patterns of low-ranking females is limited by avoidance of higher rank females, making the former more susceptible to ecological variability [122].

Other results also point to a higher expression of repulsive associations in the wet season of 2013, coinciding with the smallest core areas of all the seasons analyzed. The correlation between subgroup size and dyadic association decreased in this period, presumably the season most prone to reflect the pattern related to passive associations based on the results discussed before. However, the correlation in the wet season of 2013 fitted the prediction for active associations better than in the dry season. In addition, the permutation tests for non-random associations, suggest an increase in associations that occurred less than expected by chance from the dry to the wet season of 2013. Furthermore, of the seasons analyzed, wet 2013 had the highest number of repulsive associations. As already noted, all these associations involved male-female dyads, which consistently had lower association values than same-sex pairs, in accordance with the sex-segregated pattern described for spider monkeys [31].

Sex segregation has been well documented in *Ateles* spp., suggesting that different influences underlie the movement decisions of each sex class [31,46,71,79,89,124,125] and showing that intersexual encounters often involve male aggression towards females [80,81,115,126,127]. Previously reported differences in the socio-spatial patterns related to sex, are consistent with our observations of a tendency of males to have larger core areas than females, although only significantly during the dry season of 2014. Additionally, males showed more stable dyadic association values and average subgroup sizes, suggesting they were less influenced by seasonal shifts in fruit availability, as posed by the socio-ecological model [80]. Since males are usually expected to invest in territorial and/or female defense, the notable increase in their core areas during the dry season of 2014 could reflect space-use patterns from other females of the group not included in our analysis (particularly the immigrant females, as mentioned before) or activity from monkeys of other groups, but this could not be determined with the available data. Considering that our association measures were mostly based on individual co-occurrence, it is worth discussing how these results reflect active repulsion or avoidance rather than only different sexual needs and preferences. Movement patterns and space-use are considered to reflect individual preferences and choices [99]. If different space-use alone explained the low levels of association between males and females, we would expect this condition to be minimized when males concentrated their movements in areas equivalent to those of females, as observed in the wet season of 2013. Any effects of differing sexual-preferences on the rate of co-occurrence should have been mostly reflected in the association rates in 2014 when individuals were less prone to encounter others. Yet, most repulsive associations were observed in the wet season of 2013, when individuals had the highest probability of encounter due to similar spatial decisions. Therefore, individual grouping decisions seem to have acted against the high probability of random encounter. Although the highest average dyadic association value for male-female dyads was observed in this season, it was still significantly less than values for same-sex dyads as in every other season. This suggests that the high probability of random encounter in the wet season of 2013 derived from core area contraction particularly affected male-female encounters, seemingly exposing male-avoidance strategies by females, not derived from food competition. In sum, our results highlight sexual differences in space-use and indicate that, although not directly dependent on food competition, male-female avoidance can be particularly relevant in shaping the socio-spatial behavior of individuals when activities are confined to small areas that increase the probability of random encounters between males and females.

Distinctive association and space-use patterns observed in female spider monkeys have been related to reproductive status [78,115] and group tenure [61]. On our study, most females showed coincident socio-spatial behavior, as expected under a passive association scenario. However, some contrasting results observed for individual AM are worth noting. Although not distinctive in terms of her reproductive status, AM was the most recent immigrant among those analyzed. This female shared a significantly larger proportion of her core area with the rest of the group in dry *vs*. wet seasons. AM also had lower values of spatial associations than the others during both wet seasons, which altogether could indicate that she did not move towards the same areas as the rest of the group in the fruit abundant periods, possibly avoiding the area. Furthermore, dyadic association values for AM where particularly low in all seasons, also indicating less social integration. The seasonal patterns in the socio-spatial behavior of AM suggest the influence of factors other than the distribution and availability of fruit-patches which would have affected all females similarly. Previous results have highlighted the relevance of group tenure for the integration of female spider monkeys to groups and access to high quality areas of the home range [61]. The fact that individual KL, another relatively recent immigrant, also tended to have low dyadic association values, further highlights the potential role of group tenure on social integration, although KL immigrated before JA and the latter did not show similar differences in socio-spatial patterns. In order to further investigate this matter, data on the quality of associations needs to be revised including all the females of the group.

## Conclusions

Our levels of analysis framework, as depicted in Fig 1, proved useful for identifying the presence and changing influence of both passive and active associations in the socio-spatial patterns of the study group. Our results are supportive of the model for a female-dispersing egalitarian society where socio-spatial patterns are sex dependent, but influenced by processes of passive associations, most notably during food-abundant periods. At the same time, short-term attractive and repulsive processes are constantly operating, although detailed information on the quality of associations is needed to better assess the factors promoting them. Avoidance of males by females could be the prevailing driver of association patterns in conditions of high food abundance if individuals are clustered enough that random processes increase the frequency of male-female encounters. Additionally, female tenure in the group may partially explain differing levels of spatial and social integration into the group. As noted by Aureli et al. [20], ecological factors such as fruit abundance interact with social dynamics to determine socio-spatial behavior. Although links between resource availability and group membership are well known in primates, evidence is still scant on the effect of social constraints and their interplay with ecological constraints on grouping and space-use decisions in spider monkeys and other high fission-fusion dynamics species. The results of our study and the methodological approach used to discern between the processes influencing the co-occurrence of individuals contribute to our understanding of how social animals respond to changing ecological and social contexts.

## Supporting Information

S1 Fig Normalized values of the index of fruit abundance. (PDF)

S2 Fig Seasonal overlap of individual core areas. (PDF)

S3 Fig Example calculations of the group (gSGI) and individual (iSGI) spatial gregariousness indices. (PDF)

S4 Fig Core area as a function of core area overlap level per season. (PDF)

S5 Fig Average individual spatial gregariousness index (iSGI). (PDF)

S6 Fig Seasonal individual spatial gregariousness (iSGI) by sex. (PDF)

S7 Fig Individual values of the dyadic association index (a) and spatial dyadic association index (b). (PDF)

S8 Fig Random dyadic association index (R.DAI; a) and dyadic association index for observations within the core areas (UD.DAI; b). (PDF)

S9 Fig Non-random associations. (PDF)

S10 Fig Seasonal association networks. (PDF)

S1 File Scan data. Instant scan data for 11 adult spider monkeys (*Ateles geoffroyi*) from the *Otoch Ma’ax Yetel Kooh* protected area, Yucatan, Mexico.
(CSV)

S2 File Subgroup-size. Data on adult subgroup-size for all the subgroup observations including at least one adult individual during the study period.
(CSV)

S3 File Fruit abundance data. Estimates of fruit abundance from a fortnightly monitoring program of the tree species most consumed by the spider monkeys at the *Otoch Ma’ax Yetel Kooh* protected area, Yucatan, Mexico.
(CSV)

S1 Table Number of subgroup scans and days in which each of the study subjects was observed during the study period. (PDF)

S2 Table Seasonal core area size (ha) for the individuals of the study group. (PDF)

S3 Table Spatial gregariousness index (SGI). (PDF)

S4 Table Seasonal differences in average subgroup size. (PDF)

S5 Table Seasonal dyadic differences in the dyadic association index. (PDF)

S6 Table Seasonal dyadic differences in the spatial dyadic association index. (PDF)

S7 Table Results for additional association metrics. (PDF)
