# Supplementary material for: Seasonal Changes in Socio-Spatial Structure in a Group of Free-Living Spider Monkeys (Ateles geoffroyi)
Source: PLoS One. 2016 Jun 9;11(6):e0157228. doi: 10.1371/journal.pone.0157228 (PMC4900631; doi:10.1371/journal.pone.0157228)
Supplement: S1 Fig — (PDF) [file pone.0157228.s001.pdf]

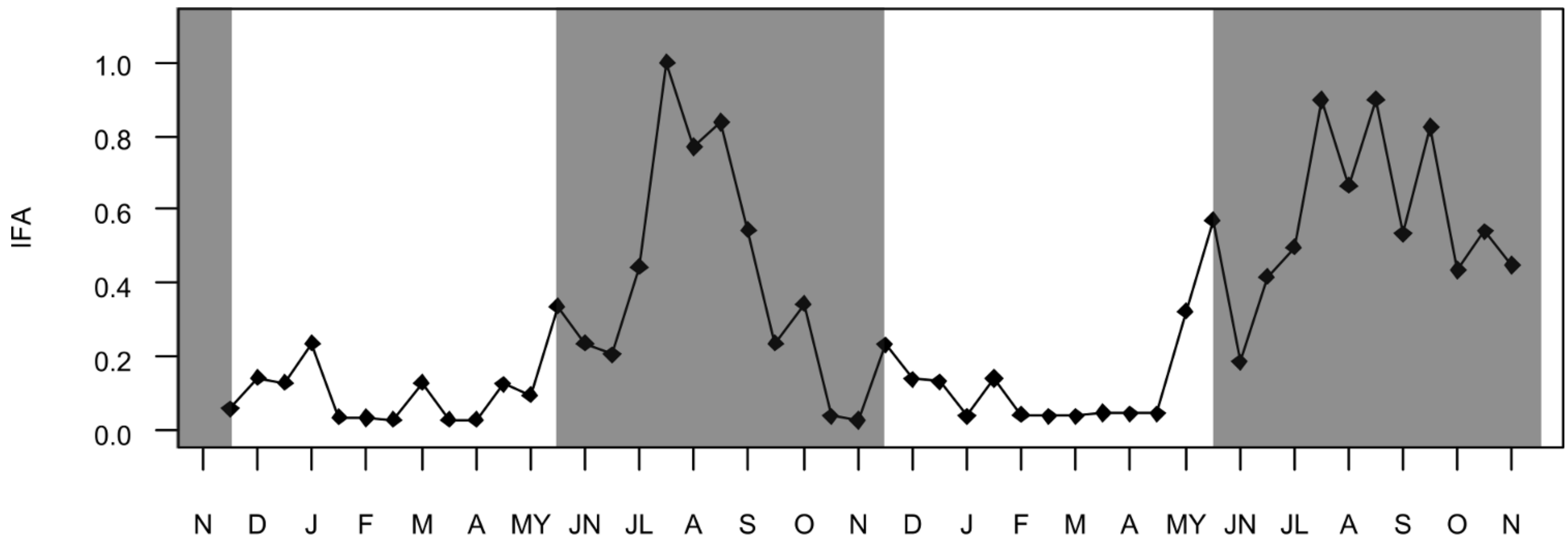

S1 Fig. Normalized values of the index of fruit abundance (IFA; black points) from mid-November 2012 to November 2014. Shaded areas highlight wet season periods (mid-May through mid-November in both years). IFA values are expressed as proportions of the maximum value obtained during the period displayed.
