# Supplementary material for: Seasonal Changes in Socio-Spatial Structure in a Group of Free-Living Spider Monkeys (Ateles geoffroyi)
Source: PLoS One. 2016 Jun 9;11(6):e0157228. doi: 10.1371/journal.pone.0157228 (PMC4900631; doi:10.1371/journal.pone.0157228)
Supplement: S3 Fig — (PDF) [file pone.0157228.s003.pdf]

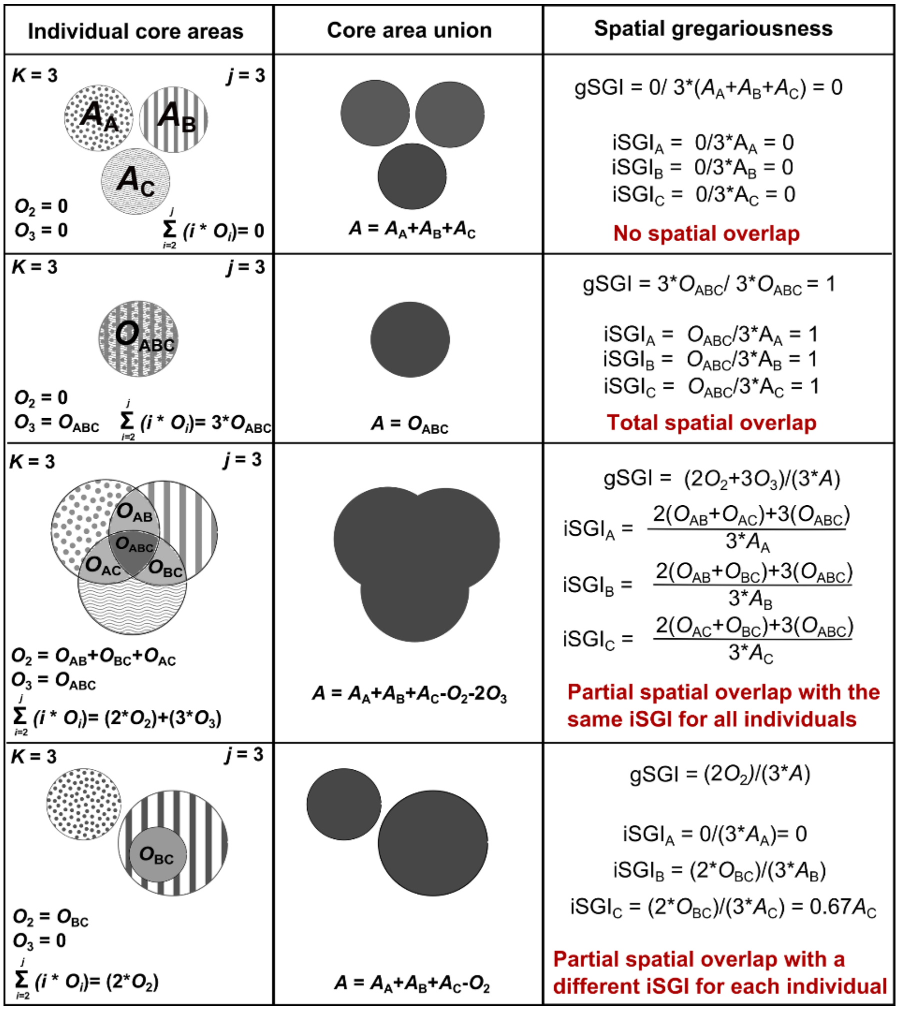

S3 Fig. Example calculations of the group (gSGI) and individual (iSGI) spatial gregariousness indices in four scenarios which differ in the level of overlap (*O*) among the core areas (CA) of three individuals (A, B and C). gSGI quantifies the clumping of individual CAs with respect to the total extent covered by the union of all core areas (CA union) following the expression $(\sum_{i=2}^{j} i * O_i)/(K * A)$ where *A* is the size of the CA union; *j* is the maximum number of overlapping individual CAs in a certain period; *i* is the number of overlapping CAs with values between 2 and *j*; *O* is the size of the area where *i* CAs overlap within the CA union; and *K* is the total number of CAs analyzed per period. Values of gSGI range between 0 and 1 where 1 indicates total spatial overlap of all possible CAs and 0 indicates no coincidence at all (i.e. completely non-overlapping CAs). iSGI quantifies how much the core area of individual *x* coincides with the rest of the CAs. It involves a formulation similar to gSGI where instead of *A*, the denominator includes the individual's core area A_x, and the overlap O_i is restricted to areas of overlap within A_x, becoming O_{ix} in the expression $\text{iSGI}_x = (\sum_{i=2}^{j} i * O_{ix})/(K * A_x)$. Values of iSGI also range between 0 and 1 where 1 indicates total spatial overlap of the individual's CA with all other possible CAs and 0 indicates no coincidence at all between that individual's CA and any other. Both indices are adapted from the index used by José-Domínguez et al. [103].
