# Supplementary material for: Seasonal Changes in Socio-Spatial Structure in a Group of Free-Living Spider Monkeys (Ateles geoffroyi)
Source: PLoS One. 2016 Jun 9;11(6):e0157228. doi: 10.1371/journal.pone.0157228 (PMC4900631; doi:10.1371/journal.pone.0157228)
Supplement: S4 Fig — (PDF) [file pone.0157228.s004.pdf]

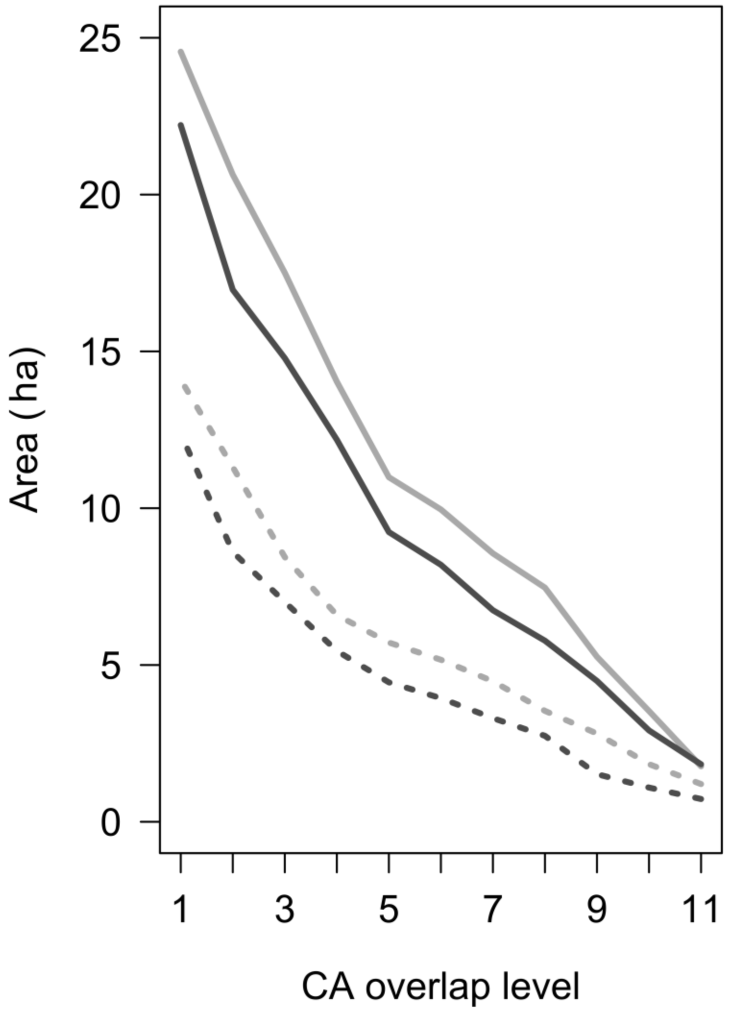

S4 Fig. Core area (CA) as a function of CA overlap level per season (light gray: dry, dark gray: wet), for 2013 (dashed lines) and 2014 (solid lines). Levels indicate the area of overlap for each corresponding number of individual CAs or more (cumulative extent). All curves have their maximum in the CA union (level 1 in the x axis) that corresponds to the total extent of the habitat used as core area by any of the 11 individuals analyzed. Similarly, the second level of overlap indicates the area where two or more CAs overlapped, and so on, up to the 11th level, which refers to the portion of the home range used as CA by all 11 individuals during the season.
