# Supplementary material for: Seasonal Changes in Socio-Spatial Structure in a Group of Free-Living Spider Monkeys (Ateles geoffroyi)
Source: PLoS One. 2016 Jun 9;11(6):e0157228. doi: 10.1371/journal.pone.0157228 (PMC4900631; doi:10.1371/journal.pone.0157228)
Supplement: S5 Fig — (PDF) [file pone.0157228.s005.pdf]

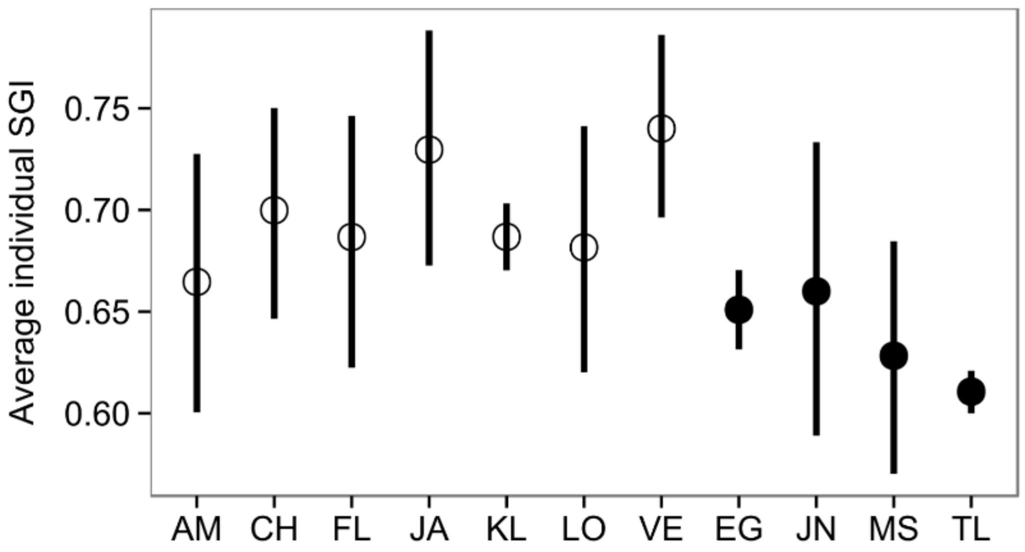

S5 Fig. Average individual spatial gregariousness index (SGI) for the females (empty circles) and males (full circles) of the study group. 95% confidence intervals based on 1000 bootstrapped replications of the original data.
