# Supplementary material for: Seasonal Changes in Socio-Spatial Structure in a Group of Free-Living Spider Monkeys (Ateles geoffroyi)
Source: PLoS One. 2016 Jun 9;11(6):e0157228. doi: 10.1371/journal.pone.0157228 (PMC4900631; doi:10.1371/journal.pone.0157228)
Supplement: S6 Fig — (PDF) [file pone.0157228.s006.pdf]

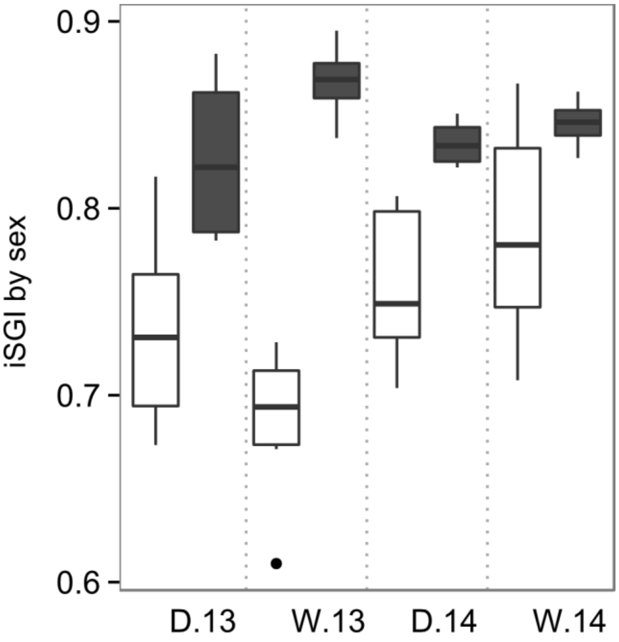

S6 Fig. **Seasonal individual spatial gregariousness (iSGI) by sex** for females (white) and males (dark gray; D.13: dry 2013, W.13: wet 2013, D.14: dry 2014 & W.14: wet 2014).
