# Supplementary material for: Seasonal Changes in Socio-Spatial Structure in a Group of Free-Living Spider Monkeys (Ateles geoffroyi)
Source: PLoS One. 2016 Jun 9;11(6):e0157228. doi: 10.1371/journal.pone.0157228 (PMC4900631; doi:10.1371/journal.pone.0157228)
Supplement: S7 Fig — (PDF) [file pone.0157228.s007.pdf]

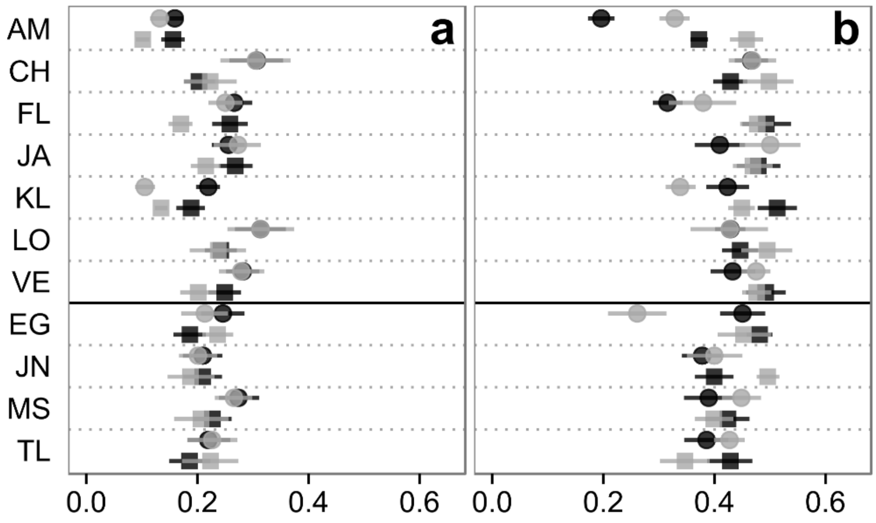

S7 Fig. Individual values of the dyadic association index (a) and spatial dyadic association index (b) for the dry (light gray) and wet (dark gray) seasons of 2013 (circles) and 2014 (squares). The solid black line divides females (top) from males (bottom) and dotted lines separate the values for each individual.
