# Supplementary material for: Seasonal Changes in Socio-Spatial Structure in a Group of Free-Living Spider Monkeys (Ateles geoffroyi)
Source: PLoS One. 2016 Jun 9;11(6):e0157228. doi: 10.1371/journal.pone.0157228 (PMC4900631; doi:10.1371/journal.pone.0157228)
Supplement: S8 Fig — (PDF) [file pone.0157228.s008.pdf]

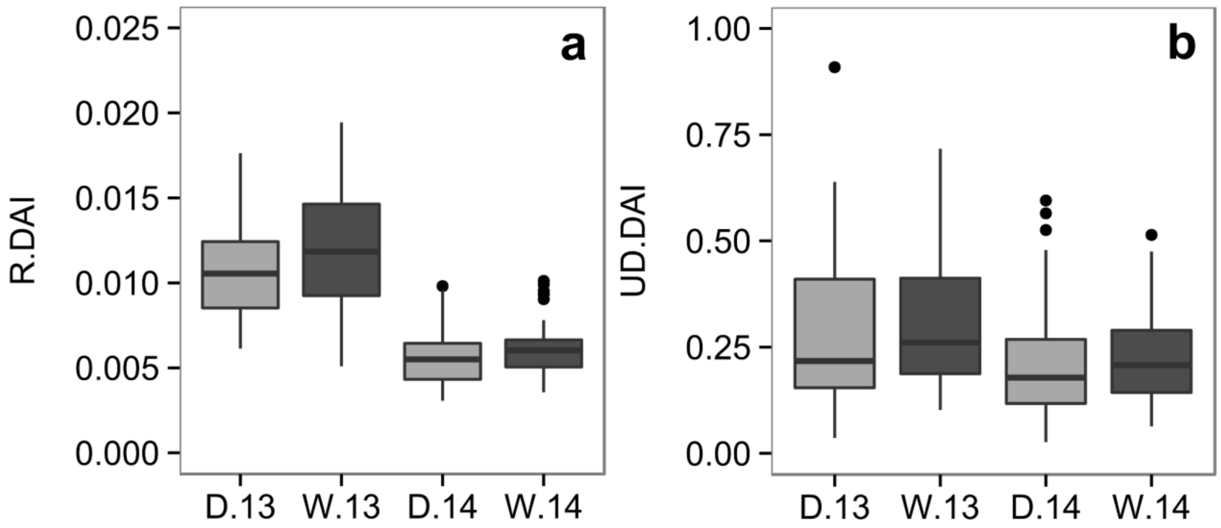

S8 Fig. Random dyadic association index (R.DAI; **a**) and dyadic association index for observations within the core areas (UD.DAI; **b**) for the dry (light gray) and wet (dark gray) seasons of 2013 and 2014. Points are observations outside 1.5 times the interquartile range above the upper quartile and bellow the lower quartile (D.13: dry 2013, W.13: wet 2013, D.14: dry 2014 & W.14: wet 2014).
