# Supplementary material for: Seasonal Changes in Socio-Spatial Structure in a Group of Free-Living Spider Monkeys (Ateles geoffroyi)
Source: PLoS One. 2016 Jun 9;11(6):e0157228. doi: 10.1371/journal.pone.0157228 (PMC4900631; doi:10.1371/journal.pone.0157228)
Supplement: S9 Fig — (PDF) [file pone.0157228.s009.pdf]

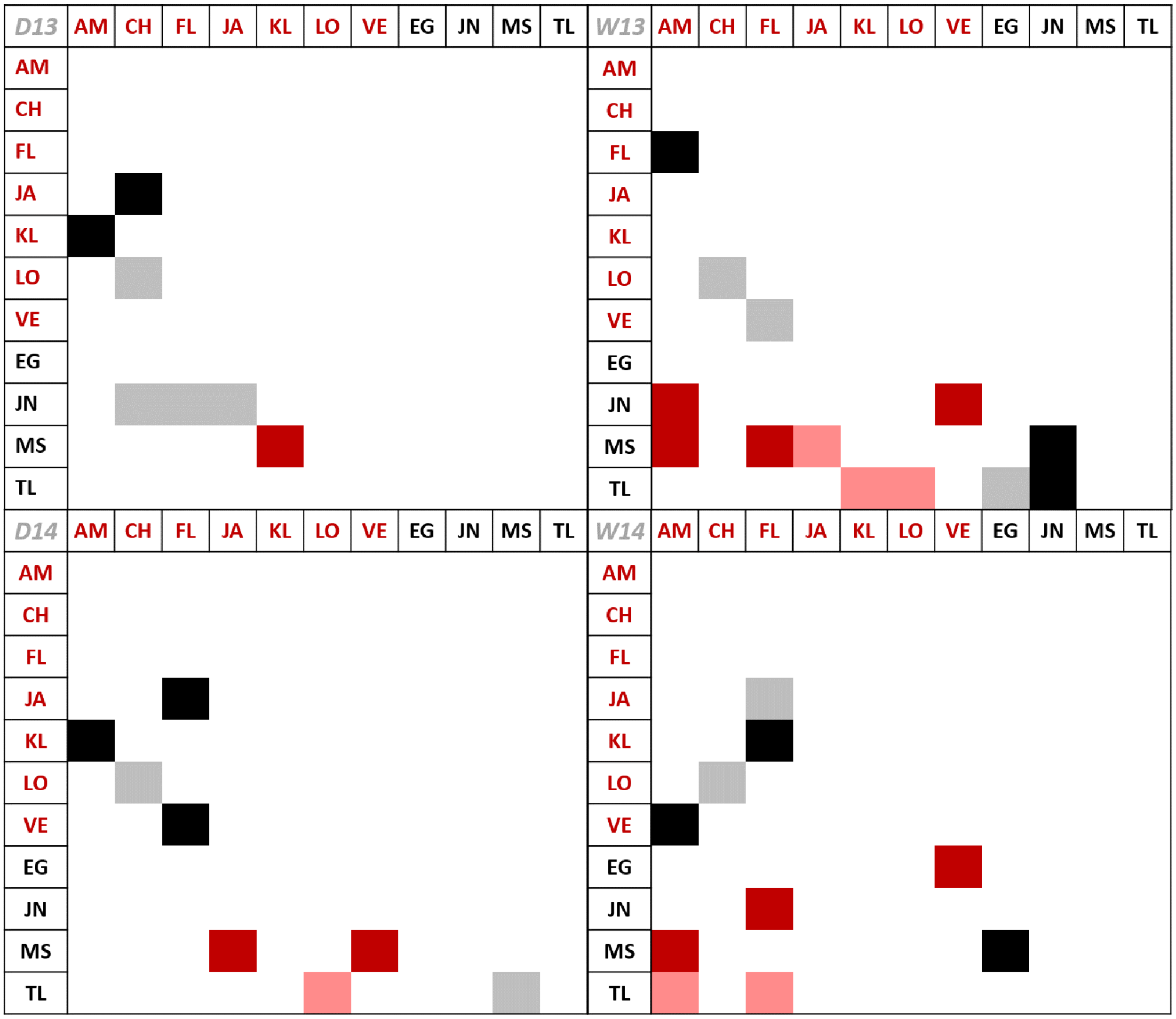

S9 Fig. Non-random associations identified each season by permutation tests using the dyadic association index values (adjusted for gregariousness) as the measure of association. Columns and rows represent the 11 individuals analyzed, identifying females (red letters) and males (black letters) by two letter codes. Associations identified in both tests (with and without LO) are shown in dark colors (black: attractive; red: repulsive) and those resulting only in one of the tests have paler tones (gray: attractive; pink: repulsive). Labels in italics indicate the season analyzed (D13: dry 2013; W13: wet 2013; D14: dry 2014 and W14: wet 2014).
