# Supplementary material for: Seasonal Changes in Socio-Spatial Structure in a Group of Free-Living Spider Monkeys (Ateles geoffroyi)
Source: PLoS One. 2016 Jun 9;11(6):e0157228. doi: 10.1371/journal.pone.0157228 (PMC4900631; doi:10.1371/journal.pone.0157228)
Supplement: S10 Fig — (PDF) [file pone.0157228.s010.pdf]

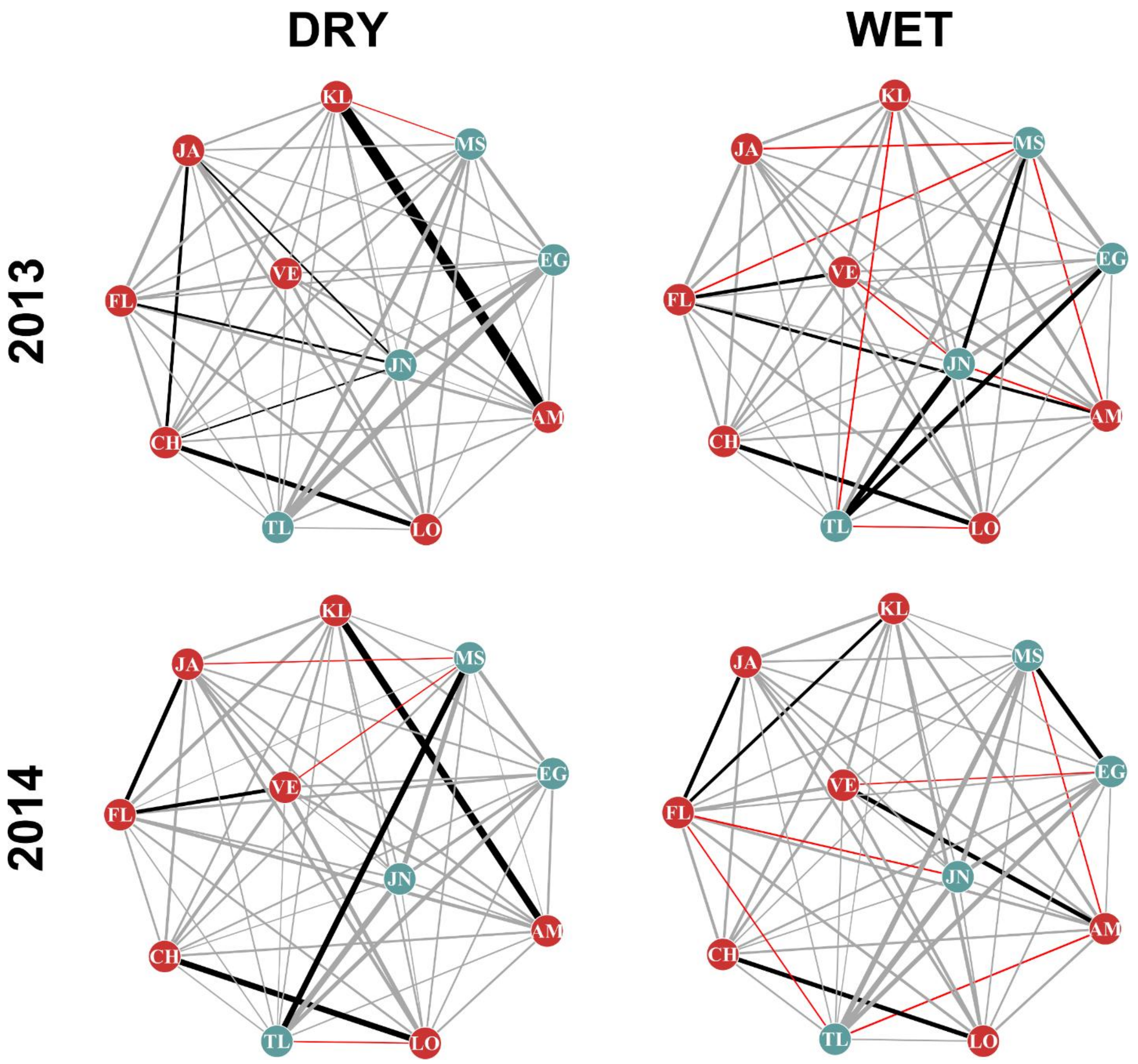

S10 Fig. Association networks for the dry (left column) and wet (right column) seasons of 2013 (top) and 2014 (bottom). Nodes indicate females (red) or males (blue) and link width is proportional to the value of the dyadic association index for each dyad. Dyads which were identified as non-random in either of the two permutation tests conducted (with and without LO) are shown in black if they occurred more (attractive) and red if they occurred less (repulsive) than expected by chance, and are otherwise shown in light gray.
