# Supplementary material for: Seasonal Changes in Socio-Spatial Structure in a Group of Free-Living Spider Monkeys (Ateles geoffroyi)
Source: PLoS One. 2016 Jun 9;11(6):e0157228. doi: 10.1371/journal.pone.0157228 (PMC4900631; doi:10.1371/journal.pone.0157228)
Supplement: S1 Table — (PDF) [file pone.0157228.s014.pdf]

S1 Table. Number of subgroup scans and days in which each of the study subjects was observed during the study period. Ages indicate number of years of life by January 2013 for males (M) and LO (natal female). For females (F), they represent a minimum age estimate assuming they were eight years old when they were first sighted with an offspring, and an inter-birth interval of 3 years. Three females already residing with the study group by 1997 (*) all had one or more offspring when first identified. For them, tenure only represents the time since they were first identified. When the age/tenure value is the same it means that the individual was born in the group.

			Subgroup scans/days observed				Total	
**Individual**	**Age/tenure (years)**	**Sex**	**DRY 2013**	**WET 2013**	**DRY 2014**	**WET 2014**	**Scans**	**Days**
AM	12/5	F	176/29	211/34	184/30	195/41	766	134
CH*	32/18	F	492/57	512/68	508/61	219/42	1731	228
EG	9/9	M	187/30	387/52	381/58	219/40	1174	180
FL*	26/18	F	344/48	391/53	262/42	280/51	1277	194
JA	12/8	F	412/50	389/56	402/59	308/52	1511	217
JN	9/9	M	219/31	304/45	311/45	251/49	1085	170
KL	16/10	F	152/22	343/46	243/42	267/43	1005	153
LO	11/11	F	494/59	550/64	513/63	274/45	1831	231
MS	5/5	M	332/42	440/59	371/51	252/42	1395	194
TL	11/11	M	223/35	339/47	361/51	231/40	1154	173
VE*	29/18	F	408/54	447/61	358/55	286/49	1499	219
**Total scans/days**			1030/73	1366/91	1707/91	1018/70	4916	325
