# Supplementary material for: Seasonal Changes in Socio-Spatial Structure in a Group of Free-Living Spider Monkeys (Ateles geoffroyi)
Source: PLoS One. 2016 Jun 9;11(6):e0157228. doi: 10.1371/journal.pone.0157228 (PMC4900631; doi:10.1371/journal.pone.0157228)
Supplement: S2 Table — (PDF) [file pone.0157228.s015.pdf]

**S2 Table. Seasonal core area size (ha) for the individuals of the study group.** Data includes averages and standard deviations (S.D.) per individual and sex class (F: female; M: male).

| ID | SEX | DRY 2013 | WET 2013 | DRY 2014 | WET 2014 | AVERAGE ±S.D. |
|---|---|---|---|---|---|---|
| AM | F | 3.7 | 5.3 | 11.9 | 7.2 | 7.0 ±3.6 |
| CH | F | 6.2 | 4.5 | 8.6 | 12.6 | 8.0 ±3.5 |
| FL | F | 8.1 | 4.0 | 10.9 | 7.3 | 7.6 ±2.8 |
| JA | F | 6.2 | 5.3 | 8.2 | 6.8 | 6.6 ±1.2 |
| KL | F | 4.0 | 4.1 | 10.0 | 9.2 | 6.8 ±3.3 |
| LO | F | 7.3 | 5.8 | 8.4 | 13.6 | 8.8 ±3.4 |
| VE | F | 4.3 | 4.5 | 9.1 | 8.1 | 6.5 ±2.3 |
| EG | M | 4.8 | 4.6 | 15.2 | 10.6 | 8.8 ±5.1 |
| JN | M | 5.1 | 4.6 | 15.4 | 9.6 | 8.7 ±5.0 |
| MS | M | 8.8 | 3.6 | 14.3 | 10.2 | 9.2 ±4.4 |
| TL | M | 6.8 | 5.0 | 12.4 | 10.5 | 8.7 ±3.3 |
| **Females (average ± s.d)** | | **5.7 ±1.7** | **4.8 ±0.7** | **9.6 ±1.4** | **9.3 ± 2.7** | **7.3 ±2.7** |
| **Males (average ± s.d)** | | **6.4 ±1.8** | **4.5 ±0.6** | **14.3 ±1.4** | **10.2 ±0.5** | **8.8 ± 4.1** |
| **Total (average ± s.d)** | | **5.9 ±1.7** | **4.6 ±0.6** | **11.3 ±2.7** | **9.6 ±2.2** | **7.9 ±3.3** |
