# Supplementary material for: Seasonal Changes in Socio-Spatial Structure in a Group of Free-Living Spider Monkeys (Ateles geoffroyi)
Source: PLoS One. 2016 Jun 9;11(6):e0157228. doi: 10.1371/journal.pone.0157228 (PMC4900631; doi:10.1371/journal.pone.0157228)
Supplement: S3 Table — (PDF) [file pone.0157228.s016.pdf]

S3 Table. Spatial gregariousness index (SGI) based on the core area union (Group SGI) and on individual core areas (Individual SGI), presenting averages and standard deviation (SD) for all individuals together, for only females and only males.

SGI	DRY 2013	WET 2013	DRY 2014	WET 2014
**Group SGI**	0.50	0.50	0.53	0.54
**Individual SGI (average** ± S.D.**)**	0.68 ±0.068	0.65 ±0.046	0.69 ±0.076	0.68 ±0.065
**Individual SGI: females (average** ± S.D.**)**	0.70 ±0.055	0.64 ±0.047	0.74 ±0.046	0.72 ±0.058
**Individual SGI: males (average** ± S.D.**)**	0.66 ±0.090	0.65 ±0.049	0.60 ±0.017	0.63 ±0.019
