# Supplementary material for: Seasonal Changes in Socio-Spatial Structure in a Group of Free-Living Spider Monkeys (Ateles geoffroyi)
Source: PLoS One. 2016 Jun 9;11(6):e0157228. doi: 10.1371/journal.pone.0157228 (PMC4900631; doi:10.1371/journal.pone.0157228)
Supplement: S4 Table — (PDF) [file pone.0157228.s017.pdf]

When examining the size of subgroups of different sexual composition (all females: F-F, all males: M-M and mixed-sex: M-F), M-F subgroups were significantly larger than F-F and M-M subgroups in all seasons while F-F subgroups were significantly larger than M-M subgroups in the wet season of 2013, but smaller in the dry season of 2014 and not significantly different in either the dry season of 2013 or the wet season of 2014.

S4 Table. Seasonal differences in average subgroup size depending on the sexual composition of the subgroup. Kruskal-Wallis tests indicated differences in subgroup size in all seasons (**DRY 2013**, KW: x^2= 260.3, df =2, P<0.0001; **WET 2013**, KW: x^2= 242.4, df=2, P<0.0001; **DRY 2014**, KW: x^2= 271.5, df=2, P<0.0001; **WET 2014**, KW: x^2= 147.3, df=2, P<0.0001). Each row presents the results from comparisons between subgroup-types per season using Mann-Whitney U tests. Only significant differences are presented (P_{adj}<0.05 after Bonferroni adjustment for multiple comparisons). Each result indicates if the first subgroup-type (as mentioned in the Subgroup-type column) had larger (>) or smaller (<) SGS than the second.

Subgroup-types	DRY 2013	WET 2013	DRY 2014	WET 2014
F-F *vs.* M-F	< U=26187.5, $n_{F\text{-}F/M\text{-}F}$=374/372, P_{adj}<0.0001	< U=64064, $n_{F\text{-}F/M\text{-}F}$=427/541, P_{adj}< 0.0001	< U=55786.5, $n_{F\text{-}F/M\text{-}F}$=546/468, P_{adj}< 0.0001	< U=33423.5, $n_{F\text{-}F/M\text{-}F}$=346/351, P_{adj}<0.0001
M-M *vs.* M-F	< U=6079.5, $n_{M\text{-}M/M\text{-}F}$=100/372, P_{adj}<0.0001	< U=13272.5, $n_{M\text{-}M/M\text{-}F}$=155/541, P_{adj}< 0.0001	< U=24822.5, $n_{M\text{-}M/M\text{-}F}$=194/468, P_{adj}< 0.0001	< U=9633.5 $n_{M\text{-}M/M\text{-}F}$=128/351, P_{adj}<0.0001
F-F *vs.* M-M	-	> U=40458.5, $n_{F\text{-}F/M\text{-}M}$=427/155, P_{adj}<0.0001	< U=41644, $n_{F\text{-}F/M\text{-}M}$=546/194, P_{adj}<0.0001	-
