# Supplementary material for: Seasonal Changes in Socio-Spatial Structure in a Group of Free-Living Spider Monkeys (Ateles geoffroyi)
Source: PLoS One. 2016 Jun 9;11(6):e0157228. doi: 10.1371/journal.pone.0157228 (PMC4900631; doi:10.1371/journal.pone.0157228)
Supplement: S5 Table — (PDF) [file pone.0157228.s018.pdf]

S5 Table. **Seasonal dyadic differences in the dyadic association index** depending on the sexual composition of the dyad: female-female (FF), male-male (MM) and male-female (MF). Each row presents the results from comparisons between dyad-types per season using Mann-Whitney U tests. For significant differences between dyad-types (P_{adj}<0.05 after Bonferroni adjustment for multiple comparisons), each result indicates if the first dyad-type (as mentioned in the Dyad-type column) had higher (>) or lower (<) values of the index than the second.

Dyad-types	DRY 2013	WET 2013	DRY 2014	WET 2014
FF *vs*. MF	U=406, $n_{FF/MF}$=21/28, P_{adj}=0.07	> U =547, $n_{FF/MF}$=21/28, **P_{adj}<0.0001**	> U =444, $n_{FF/MF}$=21/28, **P_{adj}=0.006**	> U =559, $n_{FF/MF}$=21/28, **P_{adj}<0.0001**
MM *vs*. MF	> U =165, $n_{MM/MF}$=6/28, **P_{adj}< 0.0001**	> U =168, $n_{MM/MF}$=6/28, **P_{adj}< 0.001**	> U =168, $n_{MM/MF}$=6/28, **P_{adj}< 0.0001**	> U =168, $n_{MM/MF}$=6/28, **P_{adj}< 0.0001**
FF *vs*. MM	U =42, $n_{FF/MM}$=21/6, P_{adj}=0.7	U =27, $n_{FF/MM}$=21/6, P_{adj}=0.1	< U =13, $n_{FF/MM}$=21/6, **P_{adj}=0.006**	U =28, $n_{FF/MM}$=21/6, P_{adj}=0.1
