# Supplementary material for: Seasonal Changes in Socio-Spatial Structure in a Group of Free-Living Spider Monkeys (Ateles geoffroyi)
Source: PLoS One. 2016 Jun 9;11(6):e0157228. doi: 10.1371/journal.pone.0157228 (PMC4900631; doi:10.1371/journal.pone.0157228)
Supplement: S6 Table — (PDF) [file pone.0157228.s019.pdf]

S6 Table. **Seasonal dyadic differences in the spatial dyadic association index** depending on the sexual composition of the dyad: female-female (FF), male-male (MM) and male-female (MF). Each row presents the results from comparisons between dyad-types per season using Mann-Whitney U tests. For significant differences between dyad-types (P_{adj}<0.05 after Bonferroni adjustment for multiple comparisons), each result indicates if the first dyad-type (as mentioned in the Dyad-type column) had higher (>) or lower (<) values of the index than the second.

Dyad-types	DRY 2013	WET 2013	DRY 2014	WET 2014
FF *vs*. MF	U =350, $n_{FF/MF}$=21/28, P_{adj}=0.8	U =303, $n_{FF/MF}$=21/28, P_{adj}=1	> U =487, $n_{FF/MF}$=21/28, $\boldsymbol{P_{adj}}$**=0.0001**	> U =538, $n_{FF/MF}$=21/28, $\boldsymbol{P_{adj}}$**<0.0001**
MM *vs*. MF	U =81, $n_{MM/MF}$=6/28, P_{adj}=1	U =111, $n_{MM/MF}$=6/28, P_{adj}=0.7	U =131, $n_{MM/MF}$=6/28, P_{adj}=0.09	> U =168, $n_{MM/MF}$=6/28, $\boldsymbol{P_{adj}}$**<0.0001**
FF *vs*. MM	U =70, $n_{FF/MM}$=21/6, P_{adj}=1	U =46, $n_{FF/MM}$=21/6, P_{adj}=1	U =50, $n_{FF/MM}$=21/6, P_{adj}=1	U =41, $n_{FF/MM}$=21/6, P_{adj}=0.6
