# Supplementary material for: Seasonal Changes in Socio-Spatial Structure in a Group of Free-Living Spider Monkeys (Ateles geoffroyi)
Source: PLoS One. 2016 Jun 9;11(6):e0157228. doi: 10.1371/journal.pone.0157228 (PMC4900631; doi:10.1371/journal.pone.0157228)
Supplement: S7 Table — (PDF) [file pone.0157228.s020.pdf]

S7 Table. Results for additional association metrics. Seasonal correlation between subgroup-size (SGS) and the dyadic association index (DAI) using Kendall's coefficient (*Kτ*); coefficient of variation for the dyadic association index (CV DAI); social network metrics (seasonal averages for individual values of strength and clustering coefficient, showing standard errors (S.E.) derived from 1000 bootstrapped replicates); and results of the permutation tests for non-random associations including all 11 individuals (_1), the repetition excluding LO, the adult natal female (_2) and the combination of all different associations detected by both tests (_1 & _2).

	SEASON			
	**DRY 2013**	**WET 2013**	**DRY 2014**	**WET 2014**
**Correlation SGS-DAI (K_τ, *P< 0.0001*)**	-0.36	-0.66	-0.64	-0.44
**CV DAI**	0.64	0.49	0.65	0.49
**Average strength (S.E.)**	8.73 (0.55)	8.71 (0.38)	8.50(0.34)	9.01(0.27)
**Average clustering coefficient (S.E.)**	0.58 (0.01)	0.62 (0.01)	0.54 (0.01)	0.64 (0.00)
**Non-random associations_1 ***	4 (3/1)	11 (5/6)	9 (5/3)	8 (5/3)
**Non-random associations_2 ***	6 (5/1)	9 (4/5)	5 (3/2)	8 (3/5)
**Non-random associations _1 & _2 ***	7(6/1)	13 (6/7)	8 (5/3)	10(5/5)

* Total (attractive/repulsive)
